# Supplementary material for: Therapist-supported online cognitive therapy for post-traumatic stress disorder (PTSD) in young people: protocol for an early-stage, parallel-group, randomised controlled study (OPTYC trial)
Source: BMJ Open. 2022 Mar 21;12(3):e054852. doi: 10.1136/bmjopen-2021-054852 (PMC8938692; doi:10.1136/bmjopen-2021-054852)
Supplement: Supplementary data [file bmjopen-2021-054852supp001.pdf]

Checklist: World Health Organization Trial Registration Data Set

Data category	Information
Primary registry and trial identifying number	ISRCTN 16876240
Date of registration in primary registry	06/07/20
Secondary identifying numbers	N/A
Source(s) of monetary or material support	Medical Research Council UK
Primary sponsor	Joint Institute of Psychiatry, Psychology & Neuroscience and the South London and Maudsley NHS Foundation Trust.
Secondary sponsor(s)	N/A
Contact for public queries	Dr Patrick Smith, 020 7848 0506, patrick.smith@kcl.ac.uk
Contact for scientific queries	Dr Patrick Smith, as above
Public title	Online post-traumatic stress disorder treatment for young people and their carers
Scientific title	As above
Countries of recruitment	UK
Health condition(s) or problem(s) studied	Post-Traumatic Stress Disorder (PTSD)
Intervention(s)	Internet delivered Cognitive Therapy for PTSD in Young People (iCT-PTSD-YP)
Key inclusion and exclusion criteria	Young people:<br>1. Aged 12-17 years old<br>2. Main presenting problem is PTSD (diagnosed using CAPS-5-CA) and there is a not a co-morbid problem that would preclude treatment of PTSD<br>3. PTSD symptoms relate to a single trauma<br>4. Participant has access to compatible smartphone or larger computing device (e.g. laptop, desktop computer, iPad) with internet access and to a safe and confidential space in which to engage in iCT<br>5. Participant speaks English to a level that allows therapy without the need for an interpreter, and reads English to a level that allows independent use of iCT<br><br>Parents or carers:<br>1. Parent or carer of a young person who meets all of the inclusion criteria above and none of the exclusion criteria below<br>2. Parent or carer speaks English to a level that allows participation in therapy without the need for an interpreter, and reads English to a level that allows independent use of iCT<br>3. Parent or carer has access to compatible smartphone or larger computing device (e.g. laptop, desktop computer, iPad) with internet access

Study type	Two-arm parallel-group single-blind (outcome assessor) early-stage randomized controlled trial
Date of first enrolment	25/08/20
Target sample size	34
Recruitment status	recruiting
Primary outcome(s)	Feasibility<br>As this is an early-stage trial, the primary outcomes are feasibility outcomes and adherence metrics. Feasibility data on acceptability, compliance, retention, and delivery will be collected.<br><br>Clinical<br>The primary clinical outcome is presence or absence of PTSD 16 weeks after randomisation, determined by administration of a gold standard semi-structured interview by a trained reliable assessor who is blind to treatment allocation.
Key secondary outcomes	Secondary clinical outcomes are continuous scores on a battery of reliable and valid questionnaires measuring severity of PTSD, anxiety, and depression, completed by young people and carers.
