# Supplementary material for: Therapist-supported online cognitive therapy for post-traumatic stress disorder (PTSD) in young people: protocol for an early-stage, parallel-group, randomised controlled study (OPTYC trial)
Source: BMJ Open. 2022 Mar 21;12(3):e054852. doi: 10.1136/bmjopen-2021-054852 (PMC8938692; doi:10.1136/bmjopen-2021-054852)
Supplement: Supplementary data [file bmjopen-2021-054852supp002.pdf]

Online PTSD Treatment for Young People & Carers

Version 1.0 01-Jul-19 Centre: REC Number: 19/LO/1357 IRAS ID: 262807

**PARTICIPANT CONSENT FORM**

**This consent form is for <u>young people aged 16+</u>**

**Online PTSD treatment for Young People and their Carers (OPTYC): RCT**

**Dr Patrick Smith**

**Please initial box**

1. I confirm that I have read the information sheet dated 06/05/2020 (version 1.2) for the above study. I have had the opportunity to consider the information, ask questions and have had these answered satisfactorily. ☐

2. I understand that my participation is voluntary and that I am free to withdraw at any time without giving any reason, without my medical care or legal rights being affected. ☐

3. I understand that relevant data collected during the study may be looked at by individuals from King's College London, from regulatory authorities or from the NHS Trust, where it is relevant to my taking part in this research. I give permission for these individuals to have access to my records. ☐

4. I understand that the information collected about me will be used to support other research in the future, and may be shared anonymously with other researchers. ☐

5. I understand that my relevant confidential information will be disclosed to appropriate professionals, including my GP, if a clinical or research worker on the study becomes concerned about my own, or someone else's safety. ☐

6. I agree to my General Practitioner being informed of my participation in the study and being involved in the study, including any necessary exchange of information about me between my GP and the research team. ☐

7. I understand that the information held and maintained by South London and Maudsley NHS Foundation Trust [to be localised] may be used to help contact me or provide information about my health status. ☐

8. I consent to the recording of an interview with me being made and kept on videotape/audiotape. I understand that this recording may be used for purposes of this research project.

9. I agree to take part in the above study. ☐

| | | |
|---|---|---|
| ___________________________ | _________________ | _____________________ |
| Name of Participant | Date | Signature |
| ___________________________ | _________________ | _____________________ |
| Name of Person taking consent | Date | Signature |

Online PTSD Treatment for Young People & Carers

Version 1.0 01-Jul-19 Centre: REC Number: 19/LO/1357 IRAS ID: 262807

**PARTICIPANT ASSENT FORM**

**This assent form is for young people aged 12-15**

**Please complete this form after you have read the Information Sheet or listened to an explanation about the research.**

**Online PTSD treatment for Young People and their Carers (OPTYC): RCT**
**Dr Patrick Smith**

**Please initial box**

1. I confirm that I have read the information sheet dated 06/05/2020 (version 1.2) for the above study. I have had the opportunity to consider the information, ask questions and have had these answered satisfactorily. ☐

2. I understand that my participation is voluntary. If I decide at any time during the research that I no longer wish to take part, I can tell the researchers and pull out and I don't have to give a reason. If I pull out it will not affect my medical care or legal rights. ☐

3. I understand that relevant data collected during the study may be looked at by individuals from King's College London, from regulatory authorities or from the NHS Trust, where it is relevant to my taking part in this research. I give permission for these individuals to have access to my records. ☐

4. I understand that the information collected about me will be used to support other research in the future, and may be shared anonymously with other researchers. ☐

5. I understand that my relevant confidential information will be disclosed to appropriate professionals, including my GP, if a clinical or research worker on the study becomes concerned about my own, or someone else's safety. ☐

6. I agree to my General Practitioner being informed of my participation in the study and being involved in the study, including any necessary exchange of information about me between my GP and the research team. ☐

7. I understand that the information held and maintained by South London and Maudsley NHS Foundation Trust [to be localised] may be used to help contact me or provide information about my health status. ☐



Smith P, *et al. BMJ Open* 2022; 12:e054852. doi: 10.1136/bmjopen-2021-054852

Online PTSD Treatment for Young People & Carers

Version 1.0 01-Jul-19 Centre: REC Number: 19/LO/1357 IRAS ID: 262807

8. I agree that an interview with me can be recorded and kept on videotape/audiotape. I understand that this recording may be used for purposes of this research project. ☐

9. I understand that because I am under 16 years old, I can provide my informed assent to take part in this study, but my parent/carer will also need to provide formal consent for me to take part. I agree to take part in the above study. ☐

| ________________________________ | _______________ | _____________________ |
| --- | --- | --- |
| Name of Participant | Date | Signature |

| ________________________________ | _______________ | _____________________ |
| --- | --- | --- |
| Name of Person taking consent | Date | Signature |

Online PTSD Treatment for Young People & Carers

Version 1.2 06-May-2020 Centre: REC Number: 19/LO/1357 IRAS ID: 262807

**PARENTAL CONSENT FORM**

**This consent form is for parents/carers of participants aged 12-15**

**Online PTSD treatment for Young People and their Carers (OPTYC): RCT**
**Dr Patrick Smith**

**Please initial box**

1. I confirm that I have read the information sheet v1.2 dated 06.05.2020 for the above study. I have been consulted about my child's participation in this research project. I have had the opportunity to ask questions about the study and understand what is involved. I agree to their taking part in this research. ☐

2. I understand that my child's participation is voluntary and that I can request that they are withdrawn from the study at any time without giving any reason, and without their medical care or legal rights being affected. ☐

3. I understand that relevant data collected during the study about my child, may be looked at by individuals from King's College London, from regulatory authorities or from the NHS Trust, where it is relevant to their taking part in this research. I give permission for these individuals to have access to my child's records. ☐

4. I understand that the information collected about my child will be used to support other research in the future, and may be shared anonymously with other researchers. ☐

5. I understand that my child's relevant confidential information will be disclosed to appropriate professionals, including their GP, if a clinical or research worker on the study becomes concerned about my child's, or someone else's safety. ☐

6. I agree to my child's General Practitioner being informed of their participation in the study and being involved in the study, including any necessary exchange of information about them between their GP and the research team. ☐

7. I understand that the information held and maintained by South London and Maudsley NHS Foundation Trust [to be localised] may be used to help contact my child or provide information about their health status. ☐

8. I agree for my child's assessment, and if relevant their treatment sessions, to be audio/video recorded. I understand that this recording may be used for the purposes of this research project. ☐

9. I agree for my child to take part in the above study. ☐

| ______________________ | ______________________ | ______________ | ____________________ |
| --- | --- | --- | --- |
| Your Name | Relationship to child | Date | Signature |

Online PTSD Treatment for Young People & Carers

Version 1.2 06-May-2020 Centre: REC Number: 19/LO/1357 IRAS ID: 262807

| | | |
|---|---|---|
| ___________________________ | _______________ | _________________ |
| Name of Person taking consent | Date | Signature |

Online PTSD Treatment for Young People & Carers

Version 1.0 01-Jul-19 Centre: REC Number: 19/LO/1357 IRAS ID: 262807

**PARTICIPANT CONSENT FORM FOR PARENTS/CARERS**

**This consent form is for parents/carers who wish to take part in the study**

**Online PTSD treatment for Young People and their Carers (OPTYC): RCT**
**Dr Patrick Smith**

**Please initial box**

1. I confirm that I have read the information sheet dated 06/05/2020 (version 1.2) for the above study. I have had the opportunity to consider the information, ask questions and have had these answered satisfactorily. ☐
2. I understand that my participation is voluntary and that I am free to withdraw at any time without giving any reason, without my medical care or legal rights being affected. ☐
3. I understand that relevant data collected during the study may be looked at by individuals from King's College London, from regulatory authorities or from the NHS Trust, where it is relevant to my taking part in this research. I give permission for these individuals to have access to my records. ☐
4. I understand that the information collected about me will be used to support other research in the future, and may be shared anonymously with other researchers. ☐
5. I understand that the information held and maintained by South London and Maudsley NHS Foundation Trust [to be localised] may be used to help contact me or provide information about my health status. ☐
6. I understand that my relevant confidential information will be disclosed to appropriate professionals, including my GP, if a clinical or research worker on the study becomes concerned about my own, or someone else's safety. ☐
7. I consent to the recording of an interview with me being made and kept on videotape/audiotape. I understand that this recording may be used for purposes of this research project. ☐
8. I agree to take part in the above study. ☐

| | | |
|---|---|---|
| ________________ | ________________ | ________________ |
| Name of Participant | Date | Signature |
| ________________ | ________________ | ________________ |
| Name of Person taking consent | Date | Signature |

Online PTSD Treatment for Young People & Carers

Version 1.1 06-May-2020 Centre: REC Number: 19/LO/1354 IRAS ID: 262807

**PARTICIPANT CONSENT FORM**

**This consent form is for young people aged 16+**

**Online PTSD treatment for Young People and their Carers (OPTYC): School Screening**

**Dr Patrick Smith**

**Please initial box**

1. I confirm that I have read the information sheet dated 06.05.2020 (version 1.2) for the school screening for the OPTYC study. I have had the opportunity to consider the information, ask questions and have had these answered satisfactorily.
2. I understand that my participation is voluntary and that I am free to withdraw at any time without giving any reason, without my medical care or legal rights being affected.
3. I understand that relevant data collected during the study may be looked at by individuals from King's College London, from regulatory authorities or from the NHS Trust, where it is relevant to my taking part in this research. I give permission for these individuals to have access to my records.
4. I understand that the information collected about me will be used to support other research in the future and may be shared **anonymously** with other researchers.
5. I understand that my relevant confidential information will be disclosed to my parent/carer and appropriate professionals, including my General Practitioner (GP), if a clinical or research worker on the study becomes concerned about my own, or someone else's safety.
6. I agree to my GP being informed of my participation in the study, including any necessary exchange of information about me between my GP and the research team.
7. I agree to take part in the school screening part of this study.
8. I consent to you contacting me via the details provided below.

**PLEASE TURN OVER**

Online PTSD Treatment for Young People & Carers

Version 1.1 06-May-2020 Centre: REC Number: 19/LO/1354 IRAS ID: 262807

Your Name ______________________________

Your School and Form ______________________________

Your Contact Telephone Number ______________________________

Your Home Address ______________________________

Your Email Address ______________________________

| | | |
|---|---|---|
| ______________________ | ______________ | ________________ |
| Name of Participant | Date | Signature |

**PLEASE RETURN THIS FORM TO YOUR FORM TUTOR / SCHOOL RECEPTION STAFF / VIA THE PROVIDED FREEPOST ENVELOPE**

*To be signed by member of OPTYC Team:*

| | | |
|---|---|---|
| ______________________ | ______________ | ________________ |
| Name of Person taking consent | Date | Signature |

Online PTSD Treatment for Young People & Carers

Version 1.1 06-May-2020 Centre: REC Number: 19/LO/1354 IRAS ID: 262807

**PARTICIPANT ASSENT FORM**

**This assent form is for young people aged 12-15**

**Please complete this form after you have read the Information Sheet**

**Online PTSD treatment for Young People and their Carers (OPTYC): School Screening**

**Dr Patrick Smith**

Please **initial** box

1. I confirm that I have read the information sheet dated 06.05.2020 (version 1.2) for the school screening for the OPTYC study. I have had the opportunity to consider the information, ask questions and have had these answered satisfactorily. ☐

2. I understand that my participation is voluntary. If I decide at any time during the research that I no longer wish to take part, I can tell the researchers and pull out and I don't have to give a reason. I understand that if I pull out it will not affect my medical care or legal rights. ☐

3. I understand that relevant data collected during the study may be looked at by individuals from King's College London, from regulatory authorities or from the NHS Trust, where it is relevant to my taking part in this research. I give permission for these individuals to have access to my records. ☐

4. I understand that the information collected about me will be used to support other research in the future and may be shared **anonymously** with other researchers. ☐

5. I understand that my relevant confidential information will be disclosed to my parent/carer and appropriate professionals, including my General Practitioner (GP), if a clinical or research worker on the study becomes concerned about my own, or someone else's safety. ☐

6. I agree to my GP being informed of my participation in the study, including any necessary exchange of information about me between my GP and the research team. ☐

7. I understand that because I am under 16 years old, I can provide my informed assent to take part in this school screening, but my parent/carer will also need to provide formal consent for me to take part. I agree to take part in the school screening component to the study. ☐

Version 1.1 06-May-2020 Centre: REC Number: 19/LO/1354 IRAS ID: 262807

Name of Participant ________________ Date ___________ Signature__________

*To be signed by member of OPTYC Team:*

Name of person taking consent______________ Date __________ Signature__________

Online PTSD Treatment for Young People & Carers

Version 1.1 06-May-2020 Centre: REC Number: 19/LO/1354 IRAS ID: 262807

**PARENTAL CONSENT FORM**

**This consent form is for parents/carers of participants aged 12-15**

**Online PTSD treatment for Young People and their Carers (OPTYC): School Screening**

**Dr Patrick Smith**

Please **initial** box

1. I confirm that I have been consulted about my child's participation in the '**school screening**' part of this research project. I have read the information sheet dated 06.05.2020 (version 1.2) for the school screening and have had the opportunity to ask questions about the study and understand what is involved. ☐

2. I understand that my child's participation is voluntary and that I can request that they are withdrawn from the study at any time without giving any reason, and without their medical care or legal rights being affected. ☐

3. I understand that relevant data collected during the study about my child, may be looked at by individuals from King's College London, from regulatory authorities or from the NHS Trust, where it is relevant to their taking part in this research. I give permission for these individuals to have access to my child's records. ☐

4. I understand that the information collected about my child will be used to support other research in the future and may be shared **anonymously** with other researchers. ☐

5. I understand that my child's relevant confidential information will be disclosed to appropriate professionals, including their General Practitioner (GP), if a clinical or research worker on the study becomes concerned about my child's, or someone else's safety. ☐

6. I agree to my child's GP being informed of their participation in the study, including any necessary exchange of information about them between their GP and the research team. ☐

7. I agree for my child to take part in the school screening part of this study. ☐

8. I consent to you contacting my child and me via the details provided below. ☐

**PLEASE TURN OVER**

Online PTSD Treatment for Young People & Carers

Version 1.1 06-May-2020 Centre: REC Number: 19/LO/1354 IRAS ID: 262807

Your Child's Name ______________________________________________

Your Child's School and Form ______________________________________

Your Child's Contact Telephone Number _____________________________

Your Child's Email Address ________________________________________

Your Contact Telephone Number ____________________________________

Your Home Address ______________________________________________

Your Email Address ______________________________________________

| ______________ | ______________ | ______________ | ______________ |
|---|---|---|---|
| Your Name | Relationship to child | Date | Signature |

**PLEASE RETURN THIS FORM TO YOUR CHILD'S FORM TUTOR / SCHOOL RECEPTION STAFF/ VIA THE PROVIDED PRE-PAID ENVELOPE**

*To be signed by member of OPTYC Team:*

| ______________ | ______________ | ______________ |
|---|---|---|
| Name of Person taking consent | Date | Signature |
