# Supplementary material for: Therapist-supported online cognitive therapy for post-traumatic stress disorder (PTSD) in young people: protocol for an early-stage, parallel-group, randomised controlled study (OPTYC trial)
Source: BMJ Open. 2022 Mar 21;12(3):e054852. doi: 10.1136/bmjopen-2021-054852 (PMC8938692; doi:10.1136/bmjopen-2021-054852)
Supplement: Author's manuscript [file bmjopen-2021-054852.draft_revisions.pdf]

BMJ Open

BMJ Open is committed to open peer review. As part of this commitment we make the peer review history of every article we publish publicly available.

When an article is published we post the peer reviewers' comments and the authors' responses online. We also post the versions of the paper that were used during peer review. These are the versions that the peer review comments apply to.

The versions of the paper that follow are the versions that were submitted during the peer review process. They are not the versions of record or the final published versions. They should not be cited or distributed as the published version of this manuscript.

BMJ Open is an open access journal and the full, final, typeset and author-corrected version of record of the manuscript is available on our site with no access controls, subscription charges or pay-per-view fees (http://bmjopen.bmj.com).

If you have any questions on BMJ Open's open peer review process please email info.bmjopen@bmj.com

BMJ Open

Study protocol for an early-stage parallel groups randomised controlled trial of therapist-supported online cognitive therapy for post-traumatic stress disorder (PTSD) in young people: the OPTYC trial

Journal:	*BMJ Open*
Manuscript ID	bmjopen-2021-054852
Article Type:	Protocol
Date Submitted by the Author:	28-Jun-2021
Complete List of Authors:	Smith, Patrick; King's College London Institute of Psychiatry Psychology and Neuroscience, Psychology<br>Ehlers, Anke; Oxford University, Experimental Psychology<br>Carr, Ewan ; King's College London Institute of Psychiatry Psychology and Neuroscience, Biostatistics and Health Informatics<br>Clark, David; Oxford University, Experimental Psychology<br>Dalgleish, Tim; Cambridge University, Medical Research Council Cognition and Brain Sciences Unit<br>Forbes, Gordon; King's College London Institute of Psychiatry Psychology and Neuroscience, Biostatistics and Health Informatics<br>Goldsmith, Kimberley; King's College London Institute of Psychiatry Psychology and Neuroscience, Biostatistics and Health Informatics<br>Griffiths, Helena; King's College London Institute of Psychiatry Psychology and Neuroscience, Psychology<br>Gupta, Monica; King's College London Institute of Psychiatry Psychology and Neuroscience, Psychology<br>King, Dorothy; King's College London Institute of Psychiatry Psychology and Neuroscience, Psychology<br>Miles, Sarah; King's College London Institute of Psychiatry Psychology and Neuroscience, Psychology<br>Plant, Dominic; King's College London Institute of Psychiatry Psychology and Neuroscience, Psychology<br>Yule, William; King's College London Institute of Psychiatry Psychology and Neuroscience, Psychology<br>Meiser-Stedman , Richard ; University of East Anglia, Department of Clinical Psychology & Psychological Therapies
Keywords:	MENTAL HEALTH, Child & adolescent psychiatry < PSYCHIATRY, Anxiety disorders < PSYCHIATRY

SCHOLARONE™
Manuscripts

Study protocol for an early-stage parallel groups randomised controlled trial of therapist-supported online cognitive therapy for post-traumatic stress disorder (PTSD) in young people: the OPTYC trial

Patrick Smith[1,2], Anke Ehlers[3], Ewan Carr[4], David M Clark[3], Tim Dalgleish[5,6], Gordon Forbes[4], Kim Goldsmith[4], Helena Griffiths[1], Monica Gupta[1], Dorothy King[1], Sarah Miles[1 2], Dominic T Plant[1], William Yule[1], Richard Meiser-Stedman[7]

1. Department of Psychology, Institute of Psychiatry, Psychology and Neuroscience, King's College London
2. South London & Maudsley NHS Foundation Trust
3. Department of Experimental Psychology, University of Oxford
4. Department of Biostatistics and Health Informatics, Institute of Psychiatry, Psychology and Neuroscience, King's College London
5. Medical Research Council Cognition and Brain Sciences Unit, University of Cambridge
6. Cambridgeshire and Peterborough NHS Foundation Trust
7. Department of Clinical Psychology & Psychological Therapies, Norwich Medical School, University of East Anglia

Corresponding author:

Patrick Smith, Department of Psychology, Institute of Psychiatry, Psychology and Neuroscience, King's College London, London SE5 8AF, email patrick.smith@kcl.ac.uk

Word count

4738 (excluding title page, abstract, references, figures, tables & acknowledgments)

For peer review only - http://bmjopen.bmj.com/site/about/guidelines.xhtml

For peer review only

ABSTRACT

Introduction

Post-Traumatic Stress Disorder (PTSD) is a disabling psychiatric condition that affects a significant minority of young people exposed to traumatic events. Effective face-to-face psychological treatments for PTSD exist. However, most young people with PTSD do not receive evidence-based treatment. Remotely delivered digital interventions, have potential to significantly improve treatment accessibility. Digital interventions have been successfully employed for young people with depression and anxiety, and for adults with PTSD. However, digital interventions to treat PTSD in young people have not been evaluated. The Online PTSD Treatment for Young People & Carers (OPTYC) trial will evaluate the feasibility, acceptability, and initial indications of clinical efficacy of a novel internet-delivered Cognitive Therapy for treatment of PTSD in young people (iCT-PTSD-YP).

Methods and analysis

This protocol describes a two-arm, parallel-groups, single-blind (outcome assessor), early-stage RCT, comparing iCT-PTSD-YP with a Waiting List (WL) comparator. N=34 adolescents (12-17 years old), whose primary problem is PTSD after exposure to a single traumatic event, will be recruited. Individual patient-level randomization will allocate participants in a 1:1 ratio, randomised using minimisation according to sex and baseline symptom severity. Data on feasibility and acceptability, including recruitment, adherence, retention, and adverse events, will be reported. The primary clinical outcome is PTSD diagnosis 16-weeks post-randomisation. Secondary clinical outcomes include continuous measures of PTSD, anxiety, and depression symptoms. Regression analyses will provide preliminary estimates of the effect of iCT-PTSD-YP on PTSD diagnosis, symptoms of PTSD, anxiety and depression relative to WL. Process-outcome evaluation will consider which mechanisms

For peer review only - http://bmjopen.bmj.com/site/about/guidelines.xhtml

mediate recovery. Qualitative interviews with young people, families and therapists will evaluate acceptability.

Ethics and dissemination

The study was approved by a UK Health Research Authority (HRA) Research Ethics Committee (REC; 19/LO/1354). Findings will be disseminated broadly to participants, healthcare professionals, the public, and other relevant groups. Study findings will be published in peer-reviewed journals.

Trial registration

Prospectively registered on 6 July 2020: ISRCTN 16876240

All items from the World Health Organization Trial Registration Data Set are detailed in Appendix 1

For peer review only

For peer review only - http://bmjopen.bmj.com/site/about/guidelines.xhtml

Strengths and limitations of this study

- An early-stage trial to gather data on feasibility, acceptability and initial indications of clinical efficacy of internet delivered Cognitive Therapy for PTSD in young people (iCT-PTSD-YP)
- Young people were extensively involved in designing the phone App and website
- CT-PTSD is theory-based and has demonstrated efficacy when delivered face-to-face and iCT-PTSD is effective in adults
- This trial can be delivered entirely remotely
- This early stage RCT is not powered to detect between group effects

Keywords

PTSD, young people, cognitive therapy, trial, digital mental health

Administrative information

Title

The OPTYC trial: Study protocol for an early stage randomised controlled trial of therapist-supported online cognitive therapy for post-traumatic stress disorder (PTSD) in young people

Registration

Prospectively registered: ISRCTN 16876240

Protocol

Protocol version 1.5 (April 2021)

For peer review only - http://bmjopen.bmj.com/site/about/guidelines.xhtml

Sponsor

Institute of Psychiatry, Psychology & Neuroscience (King's College London) and the South London and Maudsley NHS Foundation Trust. The funder and sponsor approved the study design and capacity to implement. Neither the funder nor the sponsor has a role in collection, management, analysis, or interpretation of data; writing of the report; or decision to submit the report for publication. Neither the funder nor the sponsor has ultimate authority over any of these activities.

For peer review only

For peer review only - http://bmjopen.bmj.com/site/about/guidelines.xhtml

INTRODUCTION

Background and rationale

Post-Traumatic Stress Disorder (PTSD) is a disabling psychiatric condition that affects a significant minority of young people exposed to traumatic events. Trauma exposure and PTSD are both prevalent among youth. Between 15-82% of youth are exposed to traumas, and between 3-8% of youth will develop PTSD by the age of 18 years [1, 2, 3], representing a significant level of morbidity for health services. For affected individuals, PTSD is highly distressing, causes marked impairments in functioning and may run a chronic course for years or decades if left untreated [4, 5].

Effective treatments for PTSD exist. Recent reviews of psychological treatments for PTSD in youth find that various forms of Trauma-Focused Cognitive Behavioural Therapy (TF-CBT) show consistently large effects in reducing PTSD symptoms and associated comorbidities [6, 7]. Cognitive Therapy for PTSD (CT-PTSD) is a form of TF-CBT developed by our group [8, 9] recommended as a first line intervention in national and international practice guidelines[10]. The treatment is theory-based, manualised, and delivered over 10-12 individual sessions. Two published RCTs [11, 12] find that CT-PTSD is acceptable to young people, and efficacious [13].

However, most young people with PTSD do not receive an effective, evidence-based treatment. The gap between community prevalence of psychiatric disorders and treatment provision for young people is well-known and longstanding [14]. In a recent population based British study, only 40% of young people with PTSD sought help from GPs or mental health practitioners and only 20% had accessed specialist mental health services in the past year [15]. Limited access to treatment may be due to multiple interacting factors including under-capacity and long waiting times for assessment and treatment in specialist Child and Adolescent Mental Health Services [16], and the burden and inconvenience to young people and families in attending face-to-face appointments in a clinic.

For peer review only - http://bmjopen.bmj.com/site/about/guidelines.xhtml

Remote delivery of psychological therapy via the internet has enormous potential to address some of these barriers, and to increase accessibility of treatment [17]. Young people have enthusiastically endorsed the potential for digital health interventions [18]. For disorders other than PTSD, digital health interventions are known to be acceptable to young people and clinically helpful. For example, Computerised Cognitive Behavioural Therapy (C-CBT) for depression demonstrates clear clinical benefit for young people [19, 20] and is now recommended by NICE [21]. Lessons have been learned about the development of digital mental health interventions including: the need for co-design with young people [22]; the active engagement of young people in therapy; and the need for continued therapist support during treatment.

Development of remotely delivered therapy for treatment of PTSD in young people lags behind that for other disorders. Jaycox and colleagues[23] report encouraging preliminary outcomes for a self-help web-based tool to augment and enhance usual school support services for trauma-exposed youth. Kasam-Adams and colleagues[24] showed that a digital intervention for preventing PTSD symptoms in injured children was feasible and clinically promising. Ruggerio and colleagues[25] found that use of a web-based psycho-education intervention for disaster-affected adolescents was associated with improvements in PTSD symptoms. However, to our knowledge, no studies have yet reported on the development or evaluation of internet-delivered TF-CBT for treatment of PTSD in children and young people. This is surprising because face to face TF-CBT is well established as an effective treatment for PTSD in youth, and work with adults shows that PTSD is a disorder which is treatable via the internet [26].

In this project we aim to address this clear gap. We have co-designed with adolescents an internet version of CT-PTSD, to be delivered via smartphone App and website, with remote therapist support. Our longer-term intention is to determine whether this approach will help to reduce the treatment gap for young people with PTSD by making an efficacious therapy more widely available. Our aim in the current early-stage trial is to gather preliminary data on feasibility, acceptability, and

For peer review only - http://bmjopen.bmj.com/site/about/guidelines.xhtml

initial signal of clinical effects of internet-delivered Cognitive Therapy for PTSD for young people (iCT-PTSD-YP), relative to a Waiting List (WL) condition. Data gathered in the current trial will be used to inform the design and size of a future scaled-up trial.

Objectives

The primary objective is to provide data on feasibility, acceptability, compliance, retention, and delivery of iCT-PTSD-YP. The secondary objective is to provide initial estimates of the effect of iCT-PTSD-YP on symptoms of PTSD, anxiety and depression relative to a WL condition.

Trial design

This study is a two-arm, parallel groups, single-blind (outcome assessor), early stage RCT, comparing iCT-PTSD-YP with a WL comparator. Individual patient-level randomization will allocate participants in a 1:1 ratio, randomised using minimisation according to sex and baseline symptom severity.

METHODS: PARTICIPANTS, INTERVENTIONS, OUTCOMES

Patient and Public Involvement

Members of the BRC Young Person's Mental Health Advisory Group (YPMHAG; 16–25 year-olds with lived experience of using mental health services) were consulted before grant submission: they provided verbal and written feedback on the research ideas. Young people were consulted at an early stage about the design of the App via a series of focus groups held in schools. Young people receiving face-to-face CT-PTSD provided feedback on initial prototypes of the App. A young person with lived experience of using mental health services is a member of the Trial Steering Committee (TSC). We will consult the YPMHAG and the TSC about our dissemination strategy.

For peer review only - http://bmjopen.bmj.com/site/about/guidelines.xhtml

Study setting

The trial will be carried out in the UK. Trial randomisation will be carried out by King's College London Clinical Trials Unit (CTU). Trial therapists will be based at King's College London and the University of East Anglia. Referrals will be sought from 14 NHS Child and Adolescent Mental Health Services (CAMHS) in London and southeast England, all of which are registered as study sites. Referrals will also be sought from secondary schools and primary care in the same region. We will offer to carry out screening surveys in schools to identify potentially eligible young people. Self-referral from anywhere in the UK is also possible via the study website.

Eligibility criteria

Young people are eligible to be included if: they are aged 12-17 years old; their main presenting problem is PTSD and there is a not a co-morbid problem that would preclude treatment of PTSD; PTSD symptoms relate to a single trauma; they speak English to a level that allows therapy without the need for an interpreter, and they read English to a level that allows independent use of iCT; they have access to a smartphone and a larger device (laptop, desktop computer, tablet) with internet access, and they have access to a safe and confidential space in which to engage in iCT. Young people are excluded if they have: brain damage; intellectual disability; Pervasive developmental disorder or neurodevelopmental disorder, as assessed by clinical interview with parents / carers; other psychiatric diagnosis that requires treatment before PTSD, determined by clinical interview and questionnaires; moderate to high risk to self; ongoing trauma-related threat; have started treatment with psychotropic medication, or changed medication, within the last 2 months; or are currently receiving another psychological treatment, as assessed in clinical interview; or previously received Trauma-Focused CBT in relation to the same traumatic event that they are currently seeking treatment for.

For peer review only - http://bmjopen.bmj.com/site/about/guidelines.xhtml

Parents or carers are eligible to be included if they: are the parent or carer of a young person who meets all of the inclusion criteria and none of the exclusion criteria above; speak English to a level that allows participation in therapy without the need for an interpreter, and read English to a level that allows independent use of iCT; and have access to a smartphone and/or larger device with internet access.

Interventions

iCT-PTSD-YP

Internet-delivered Cognitive Therapy for PTSD for young people (iCT-PTSD-YP) comprises therapist-supported online delivery of all components from our published manual of face-to-face CT-PTSD for young people [27]. Treatment aims to change problematic appraisals, update trauma memories, and change unhelpful coping responses. Treatment components are delivered in modules. There are 10 core modules for all young people (Psychoeducation about PTSD, Reclaiming life, Understanding PTSD, Developing a trauma narrative, Identifying hotspots, Updating the narrative, Working with triggers, Overcoming sense of danger, Visiting the site virtually and/or in person, Developing a blueprint) and 11 optional modules which are used according to individual need (Relaxation, Sleep, Working with images, Working with physical difference, Anger, Grief, Shame, Guilt, Self-criticism, Rumination, and Panic). Modules were co-designed with input from young people and built on the content of the modules developed for iCT-PTSD for adults [28, 29]. Modules are interactive (prompting for user action to progress through the App and requesting user text input and questionnaire responses) and include text, illustrations, audio case examples, animations, and videos. Modules are intended for independent self-study by young people. Therapists can log onto the site to view young people's progress including their text input and questionnaire responses. Young people and therapists can message each other via the App. Parents and carers are provided a separate log on to the carer version of the App. The carer version

For peer review only

For peer review only - http://bmjopen.bmj.com/site/about/guidelines.xhtml

comprises 8 modules, and the emphasis is on providing information to carers about therapy, including advice about how carers can help in young people's recovery. Carers do not have access to any information that their child inputs to the App. Modules are delivered via a progressive web App (PWA) on a smartphone or computer, hosted on a secure server. The App is not publicly available currently. For trial participants, an individual account requiring two-factor authentication log-in is created for the young person and their carer.

Therapists will be clinical psychologists or CBT therapists who have received training in face-to-face CT-PTSD, and in use of the iCT-PTSD-YP App. Therapists will have contact with young people and carers via phone or videoconferencing at least once a week for the duration of therapy. Weekly clinical supervision will be provided by a consultant clinical psychologist from the trial team.

Therapy is delivered over 12 weeks. Post treatment assessment is carried out one month after the end of treatment (i.e. at 16 weeks after randomisation).

Waiting List

Young people will be placed on a Waiting List (WL) and re-assessed 16 weeks after randomisation. Young people who require treatment at the end of the waiting period will be offered immediate iCT-PTSD-YP. WL control arms are commonly used in PTSD treatment trials[6] because natural recovery from PTSD can be substantial [30]. Use of a WL condition ensures that the effect of treatment is not overestimated, and shows whether treatment is impeding the rate of natural recovery.

Withdrawals

Participants will be withdrawn from treatment if: a current illness prevents further treatment; there is a change in the participant's condition or circumstances that in the clinician's

For peer review only - http://bmjopen.bmj.com/site/about/guidelines.xhtml

opinion justifies the discontinuation of treatment; or the participant withdraws consent for treatment. Participants who discontinue treatment for the above reasons will be invited to provide follow-up data and will remain in the trial for the purposes of data analysis. If the participant no longer wishes to be followed up to provide research data, the participant will be withdrawn entirely from the trial. The different types of withdrawal will be captured and reported.

Outcomes

The schedule for assessments is presented in Table 1.

Feasibility outcomes

We will report: (1) the number of young people referred to the trial in total and according to referral route; (2) the number of young people screened in schools, and the proportion of those who proceed to a phone call with the family; (3) the number and proportion of young people in schools scoring above cut-off on a validated screening questionnaire (CRIES-8, see below) relative to the number of young people screened in schools; (4) the number and proportion of young people in schools who score above cut-off on the screening questionnaire but decline further participation with the trial relative to those scoring above cut-off); (5) the number and proportion of young people in schools who score above cut-off on the screening and consent to further assessment but are deemed ineligible at baseline assessment relative to those deemed eligible at baseline assessment; (6) the number of assessment appointments offered to participants; (7) the number and proportion of assessment appointments attended by participants, relative to the number of appointments offered, reported by referral source; (8) reasons for not attending assessment appointments, reported by referral source; (9) the number and proportion of young people who at baseline assessment consent to participate in the trial, relative to the number who attend

For peer review only - http://bmjopen.bmj.com/site/about/guidelines.xhtml

assessment, with reasons for not consenting if known; (10) the number and proportion of young

MEASURE STUDY PERIOD

people eligible for the trial after baseline assessment, relative to the number of baseline assessments completed; (11) the number and proportion of young people who are randomised, and the proportion of consented young people who are randomised relative to the number who consented; (12) reasons for withdrawing from the trial if known; and (13) the number retained in study at 16 weeks (post-treatment) and at 38 weeks (follow-up), and the proportions of those who start treatment who are retained.

Adherence metrics

For participants allocated to iCT-PTSD-YP, we will report: (1) the number of times logged into the programme per week and in total; (2) time spent logged in per week and in total; (3) the number of modules completed in total and according to device used; (4) the number of therapist phone calls attended per week and in total, and the number of missed phone appointments; (5) time spent on phone calls per week and in total; (6) the number of messages to / from therapist per week and in total; (7) the number and proportion of young people who start treatment; (8) the number of weeks of therapy completed and (9) reasons for dropping out of treatment if known.

Acceptability outcomes

We will carry out qualitative interviews with young people, carers, and therapists to gauge acceptability of iCT-PTSD-YP, and we will summarise interview data using content analysis. We will aim for these interviews to be representative of individuals involved in the feasibility trial (young people, carers, therapists), including young people who left the study or failed to adhere to the course of treatment, to provide a full range of views. We will interview trial participants in both arms about the acceptability of the research procedures including the assessment measures and their views on randomisation.

For peer review only - http://bmjopen.bmj.com/site/about/guidelines.xhtml

	**Screen** 0-1 weeks	**Pre** 0 weeks	**Weekly** (*iCT only*)	**Mid** 0+ 6 weeks	**Post** 0+ 16 weeks	**Follow-up** 0+38 weeks (*iCT only*)
ENROLMENT						
Eligibility screen	x					
Provide study information	*x*					
Gain informed consent		*x*				
ONLINE ASSESSMENT						
DAWBA		*x*				
INTERVIEW						
DEMOGRPAHIC INTERVIEW		*x*				
CAPS-CA-5		*x*			*x*	
CGAS		*x*			*x*	
ADOLESCENT QUESTIONNAIRES						
CPSS-5		*x*			*x*	*x*
CRIES-8		*x*	*x*	*x*	*x*	*x*
RCADS-C		*x*			*x*	*x*
CPTCI		*x*		*x*	*x*	*x*
TMQQ		*x*		*x*	*x*	*x*
Rumination items		*x*		*x*	*x*	*x*
CHU-9D		*x*			*x*	*x*
Adverse events				*x*	*x*	*x*
CARER QUESTIONNAIRES						
SDQ-P		*x*			*x*	*x*
RCADS-P		*x*			*x*	*x*
CA-SUS		*x*			*x*	*x*
Adverse events				*x*	*x*	*x*
QUALITATIVE INTERVIEWS						
Adolescents					*x*	
Carers					*x*	
Therapists					*x*	

Table 1 Study schedule

Primary clinical outcome

For peer review only

Presence of PTSD according to the DSM-5 at 16 weeks post-randomisation, ascertained using the Clinician Administered PTSD Scale for DSM-5: Child and Adolescent version (CAPS-CA-5 [30]), administered by trained reliable raters, blind to treatment allocation.

Secondary clinical outcomes

Child-reported outcomes at 16 weeks post randomisation: PTSD symptom severity (continuous score) on the CAPS-CA-5 [31]; PTSD symptom severity on the Child PTSD Symptom Scale for DSM-5 (CPSS-5[32]); PTSD symptom severity on the Children's Revised Impact of Event Scale, 8-item version (CRIES-8 [33, 34]); and symptoms of depression and anxiety on the 25-item Revised Children's Anxiety and Depression Scale (RCADS[35]). Carer reported outcomes at 16 weeks post randomisation: Revised Children's Anxiety and Depression Scale – Parent version (RCADS-P [35]); and Strength & Difficulties Questionnaire – parent version (SDQ-P [36]). At 38-week follow-up for participants in the iCT-PTSD-YP only, all secondary clinical outcomes apart from the CAPS-CA-5 will be repeated.

Process measures

The cognitive model [8] on which treatment is based specifies a number of mechanisms of therapeutic change. We will test mediation via changes in appraisals, memory quality, and ruminative thinking from baseline to mid-treatment (6-weeks post randomisation) using: the Child Post Traumatic Cognitions Inventory (CPTCI [37]); the Trauma Memory Quality Questionnaire (TMQQ[38]); and the Trauma Related Rumination Questionnaire items [39].

Health economic outcomes

For peer review only - http://bmjopen.bmj.com/site/about/guidelines.xhtml

For peer review only

We will collect economic data on health utilities and resource use using the Child Health Utility Index 9D (CHU-9D [40]) and the Child & Adolescent Service Use Schedule (CA-SUS [41]), administered at baseline and 16-weeks post-randomisation.

Participant timeline

All participants will be assessed three times during the study: pre-treatment (week 0), mid-treatment (week 6 post-randomisation), and post-treatment (week 16 post-randomisation). Participants in iCT-PTSD-YP will complete a brief weekly measure of PTSD symptoms (CRIES-8) and mood (Likert scale) on the App, and a follow up assessment (week 38 post randomisation).

Sample size

We will recruit 17 participants per arm. In our previous RCTs of face-to-face CT-PTSD [11, 12] in young people, we had 4% drop-out, but we have conservatively allowed for approximately 20% drop-out, to give at least n = 14 at post-treatment in each arm. An early-stage trial of this size will be sufficient to gather meaningful feasibility data on acceptability, compliance, retention, and delivery. Power calculations are not typically used to determine sample size for feasibility studies. Therefore, we acknowledge an insufficient sample size to allow definitive between-group comparisons in this early stage RCT [42, 43].

Recruitment

Participants will be recruited via three routes (see Figure 1): (1) from school screening; (2) from NHS CAMHS teams; and (3) from primary care (GP or school referral) or self-referral. For all

For peer review only - http://bmjopen.bmj.com/site/about/guidelines.xhtml

referral routes, consent will be sought before assessment, and eligibility will be determined by the clinical assessment.

METHODS: ASSIGNMENT OF INTERVENTIONS

Allocation

Once a participant is confirmed as eligible and consenting to the study, they will be registered in the main participant database (held using the IBM-SPSS programme). Participants will be randomised to receive iCT-PTSD-YP or WL at a 1:1 ratio. Randomisation will be carried out by the King's Clinical Trials Unit (KCTU) via a web-based service utilising minimisation with a random component. Minimisation factors will be sex and baseline PTSD symptom severity assessed by the CPSS (low: <51, high: ≥51).

Blinding

All assessors of the primary and secondary clinical outcomes at follow-up at 16 weeks will be blind to trial arm allocation. Blind outcome assessors will be independent research assistants or clinical psychologists who are not part of the trial team. Assessors will be trained to standard on the CAPS-CA-5 interview, and inter-rater reliability will be assessed for 20 randomly selected interviews. The senior trial statistician (KG) will also be blind with all other members of the study team unblind to trial arm allocation. Unblinding of the senior trial statistician and the analysis of outcomes by intervention arm will occur after the initial draft of the statistical analysis report is generated.

METHODS: DATA COLLECTION, MANAGEMENT, ANALYSIS

Data collection methods

For the primary clinical outcome, the CAPS-CA clinical interview is completed on the phone or via videoconference, with symptom level responses marked on the interview form and then

For peer review only - http://bmjopen.bmj.com/site/about/guidelines.xhtml

entered into the trial database. For secondary clinical outcomes, questionnaires are completed online via a secure commercial system (Qualtrics) with responses downloaded to an electronic database and re-entered into the trial database. Feasibility outcomes are recorded by the study research assistant in the trial database. Adherence metrics are either recorded by the trial therapist in the study database or automatically captured by the App and downloaded to standard database software.

For peer review only

Data management

Participant Information will be kept confidential and managed in accordance with the Data Protection Act, GDPR policies, NHS Caldicott Guardian, The UK Policy Framework for Health and Social Care Research, and Research Ethics Committee Approval. Personally identifiable data will be collected from participants including name and contact details. This information will be stored securely and separately from all other study-generated data, which will be anonymised. Each participant will be given a unique Participant Identification Number (PIN). All feasibility and clinical outcomes for the RCT will be stored in SPSS databases against the participant PIN. These databases will be stored on a secure KCL network drive, accessible to the study team only. Databases will be stored in a version control system, such that changes made over time can be examined and recovered. All databases will be registered in the King's Data Protection Register (KDPR).

Statistical methods

A comprehensive statistical analysis plan (SAP) will be developed and agreed with the Trial Steering Committee (TSC) before any analysis is carried out. The SAP will describe statistical procedures in detail. Quantitative analyses will employ up-to-date versions of statistical software (e.g Stata or R).

For peer review only - http://bmjopen.bmj.com/site/about/guidelines.xhtml

Analysis of feasibility outcomes and adherence metrics

The feasibility outcomes and adherence metrics will be summarised with appropriate summary statistics (e.g. means and standard deviations/medians and interquartile ranges for continuous outcomes; frequencies and proportions for count outcomes). Where appropriate some feasibility outcomes will either be reported only for the iCT-PTSD-YP arm or will be reported separately by arm.

Clinical outcomes

As this is an early-stage trial designed to gather data on feasibility outcomes, it is not powered to detect between-arm differences: where between-arm differences are presented, they will be treated as exploratory and not treated as inferential. Data completeness will be summarised for clinical outcomes. All comparative analyses will primarily be conducted under the intention-to-treat (ITT) principle – all participants with a completed outcome will be included in the analysis and analysed according to the arm they were randomised to. Where deviations from ITT occur, this will be reported. We will carry out per-protocol analyses in addition to ITT, but these analyses will be treated as secondary to the ITT analysis. There will be no interim or subgroup analyses.

The primary and secondary clinical outcomes will be summarised with appropriate summary statistics by trial arm at each time point (primary, frequencies and proportions; secondary, means and standard deviations). For each outcome we will estimate the treatment effect at 16 weeks, with the appropriate 95% confidence interval. The iCT-PTSD-YP versus WL odds ratio for remission from PTSD caseness at 16 weeks post-randomisation will be assessed using logistic regression with trial arm and the minimisation variables as covariates. The iCT-PTSD-YP versus WL mean differences in secondary clinical outcomes at 16 weeks post-randomisation will be estimated using linear regression, with trial arm, baseline outcome score and minimisation variables as covariates.

For peer review only - http://bmjopen.bmj.com/site/about/guidelines.xhtml

We will carry out per-protocol analyses for the primary outcome, and the CPSS-5 and CRIES-8 secondary outcomes at 16 weeks. These will be treated as secondary to the ITT analysis. The per protocol analyses will be conducted in two populations. The first will consist of all participants with recorded outcome data who complete the minimum therapy needed to achieve clinical benefit (defined as completing at least the first six core modules (Psychoeducation about PTSD, Reclaiming life, Understanding PTSD, Developing a trauma narrative, Identifying hotspots, Updating the narrative)). The second per protocol population will consist of all participants from the first per protocol population who have additionally completed the core module, “Working with triggers”.

Process outcomes

An exploratory mediation analysis will be carried out to assess the indirect effect of treatment allocation on the primary clinical endpoint via the Child Post Traumatic Cognitions Inventory score (CPTCI), the Trauma Memory Questionnaire (TMQQ), and items relating to rumination, measured at 6 weeks post-randomisation. The total, direct, and indirect effects of treatment allocation on 16-week PTSD caseness will be estimated using the Stata paramed command[44, 45] to properly calculate effects for a binary outcome, along with associated 95% confidence intervals. Confidence intervals for the indirect effect will be estimated using the percentile bootstrap[46].

Health economics

To gauge the feasibility of collecting health economic data, data completeness will be summarised by presenting the number and proportion of complete and missing values at each time point. Efficacy will be measured using the CHU-9D measure of health-related quality of life. Data on iCT-PTSD-YP, contact time and indirect time for the intervention will be collected directly from

For peer review only - http://bmjopen.bmj.com/site/about/guidelines.xhtml

clinicians and service records. Service use estimates will be combined with standard UK sources for unit costs to estimate total costs. The cost of iCT-PTSD-YP will be directly calculated. These data will allow us to index service use and permit preliminary estimates of the potential cost-effectiveness of iCT-PTSD-YP.

Qualitative analysis

We will carry out qualitative interviews at the end of each participant's iCT-PTSD-YP. If participants drop out of treatment early, we will endeavour to interview them. The views and experiences of patients, parents or carers, and trial clinicians will be sought in order to gain a multi-perspective view of acceptability. Content analysis will be used to explore both commonalities and variations within and between these respondents. We will interview trial participants in both arms about the acceptability of the research procedures including the assessment measures and their views on randomisation. We will invite all participants to take part in qualitative interviews, until data saturation is reached.

METHODS: MONITORING

Data monitoring

Project oversight will be provided by a monthly Project Management Group (PMG) attended by all co-investigators. Trial oversight will be provided by a 6-monthly Trial Steering Committee (TSC). The TSC will review the protocol, agree the statistical analysis plan (SAP), and safeguard the interests of trial participants. The TSC will provide advice to the CI and sponsor. A separate Data Monitoring Committee (DMC) will not be convened. The TSC will monitor adverse events and adverse reactions and will convene an emergency DMC if needed.

For peer review only - http://bmjopen.bmj.com/site/about/guidelines.xhtml

Adverse events

Adverse events (AEs) are defined as any untoward occurrence in a trial participant, including events that are not necessarily caused by or related to trial procedures. Serious adverse events are defined as AEs that result in death, are life-threatening, require hospitalisation or prolong existing hospitalisation, or result in persistent or significant disability or incapacity. Some adverse events are expected in this study, and will be reported to the TSC, for example: self-harm not requiring medical attention, increase in suicidal ideation, worsening of PTSD symptoms (defined as 7-point increase in CRIES-8). Serious AEs will be reported to the Chair of the TSC, the REC, and the sponsor. Adverse events will be assessed at each assessment time point. Risk monitoring including adverse event monitoring will be carried out during clinical contact for those allocated to iCT-PTSD-YP. AEs will be monitored and recorded from randomisation to final follow-up.

ETHICS AND DISSEMINATION

Ethical approval

The study was approved by a UK Health Research Authority (HRA) Research Ethics Committee (REC; 19/LO/1354). The study is sponsored by King's College London.

Protocol amendments

We were initially funded to run an early-stage 3-arm RCT comparing iCT-PTSD-YP with face-to-face CT-PTSD and WL. The COVID-19 pandemic national lockdown was implemented before we started to recruit to the planned 3-arm trial. Restrictions in CAMHS services due to lockdown meant that we could not offer face-to-face CT-PTSD. Therefore, after consultation with the funder and the TSC we changed the design to the current 2-arm trial and received HRA and REC approval to proceed. This change was made before recruitment started, and before registration on ISRCTN.

For peer review only - http://bmjopen.bmj.com/site/about/guidelines.xhtml

Further protocol amendments will require approval from the REC, and where relevant will be passed on to the trial register.

Consent and assent

For participants aged under 16, informed consent will be provided by carers and the young person will be asked for their assent. Participants aged 16 years or older can provide informed consent without their parent or caregiver's involvement.

Confidentiality

Information with regards to participants will be kept confidential. The treating clinician and research team involved in day-to-day trial management will have access to personally identifiable data so that they can maintain contact with participants throughout the study. Participants will be assigned a study ID. All outcome data will be stored against this study ID so that data is anonymised.

Declaration of interests

Some investigators provide training in the delivery of CT-PTSD, for which they may sometimes receive payment. PS, DMC, and WY are co-authors on a published treatment manual of CT-PTSD for children and young people[29] and receive royalties from sales.

Access to data

All investigators will have access to the final trial dataset. Our intentions are to maximise the availability and sharing of our data for the benefit of the wider research community, while

For peer review only - http://bmjopen.bmj.com/site/about/guidelines.xhtml

providing for its long-term preservation and making due allowance for the potential commercial value of findings. The PMG will make the decision on whether to supply research data to a potential new researcher. Independent oversight of data access and sharing will be provided by the TSC. Data released to the wider community after publication will be fully anonymised.

Dissemination policy

There are no publication restrictions and findings will be disseminated broadly to participants, healthcare professionals, the public, and other relevant groups. The study findings will be published in peer-reviewed journals. The full trial protocol is available from PS.

DISCUSSION

PTSD in children and adolescents is a significant public health burden. Highly efficacious treatments exist but are not widely accessible. Remotely delivered iCT-PTSD has potential to facilitate a step change in improving accessibility of an evidence-based therapy for youth. The data gathered in the current trial will inform the design and size of a future scaled up trial to evaluate remotely delivered iCT-PTSD-YP.

For peer review only

Authors' contributions:

PS, DMC, TD, AE, KG, RMS, & WY designed the trial.

PS, DMC, TD, AE, HG, MG, DK, RMS, SM, DTP & WY contributed to App development and delivery

PS, DMC, EC, TD, AE, GF, KG, HG, DK, RMS, SM, & WY oversaw recruitment and data collection.

PS drafted the protocol.

All authors read and approved the final manuscript. All authors have agreed both to be personally accountable for their own contributions and to ensure that questions related to the accuracy or integrity of any part of the work, even ones in which the author was not personally involved, are appropriately investigated, resolved, and the resolution documented in the literature.

For peer review only

Competing interests.

None

Funding

OPTYC is funded by the UK Medical Research Council's Developmental Pathway Funding Scheme (Grant reference: MR/P017355/1). TD was funded by the UK Medical Research Council (Grant Reference: SUAG/043 G101400). KG receives funding from the NIHR Applied Research Collaboration South London (King's College Hospital NHS Foundation Trust).

For peer review only - http://bmjopen.bmj.com/site/about/guidelines.xhtml

REFERENCES

1. Copeland, W. E., Keeler, G., Angold, A., et al. (2007). Traumatic Events and Posttraumatic Stress in Childhood. *Arch Gen Psychiatry*, 64, 577-584.

2. Landolt, M.A., Schnyder, U., Maier, T., et al. (2013). Trauma exposure and posttraumatic stress disorder in adolescents: A national survey in Switzerland. *Journal of Traumatic Stress*. Vol.26(2), 209-216

3. McLaughlin, K. A., Koenen, K. C., Hill, E. D., et al. (2013). Trauma exposure and posttraumatic stress disorder in a national sample of adolescents. *J Am Acad Child Adolesc Psychiatry*, 52(8), 815-830

4. Morgan, L., Scourfield, J., Williams, D. et al (2003). The Aberfan disaster: 33 year follow up of survivors. *B J Psychiat*, 182, 6, 532-36

5. Yule, W., Bolton, D., Udwin, O., et al (2000). The long term psychological effects of a disaster experienced in adolescence I: incidence and course of PTSD. *J Child Psychiat & Psychol*. 41, 4, 503-11.

6. Morina, N., Koerssen, R., & Pollet, T. (2016). Interventions for children and adolescents with posttraumatic stress disorder: A meta-analysis of comparative outcome studies. *Clinical Psychology Review* 47 (2016) 41–54

For peer review only - http://bmjopen.bmj.com/site/about/guidelines.xhtml

7. Gutermann, J., Schreiber, F., Matulis, et al. (2016). Psychological Treatments for Symptoms of Posttraumatic Stress Disorder in Children, Adolescents, and Young Adults: A Meta-Analysis. *Clin Child Fam Psychol Rev*, 19(2), 77-93

8. Ehlers, A., & Clark, D. (2000). A cognitive model of posttraumatic stress disorder. *Behav Res Ther*, 38(4), 319–345.

9. Ehlers, A., Clark, D.M., Hackmann, A., et al. (2005). Cognitive therapy for PTSD: development and evaluation. *Behav Res Ther*, *43*, 413-431.

10. National Institute for Health and Care Excellence (2018). *Posttraumatic stress disorder. NICE guideline 116. https://www.nice.org.uk/guidance/ng116/resources*

11. Smith, P., Yule, W., Perrin, S., et al. (2007) Cognitive Behavior Therapy for PTSD in Children and Adolescents: A Randomized Controlled Trial. *J Am Acad Child and Adol Psychiat*, 46, 1051–1061.

12. Meiser-Stedman, R., Smith, P., McKinnon, A., et al. (2017). Cognitive therapy as an early treatment for post-traumatic stress disorder in children and adolescents: a randomized controlled trial addressing preliminary efficacy and mechanisms of action. *Journal of Child Psychology and Psychiatry*, 58(5), 623-633.

13. Mavranezouli, I., Megnin-Viggars, O., Daly, C., et al. (2019). Research Review: Psychological and psychosocial treatments for children and young people with post-traumatic stress disorder: a network meta-analysis. *Journal of Child Psychology and Psychiatry*, 61(1), 18-29.

For peer review only - http://bmjopen.bmj.com/site/about/guidelines.xhtml

14. Ford, T. Goodman, R., & Meltzer, H. (2003). Service use over 18 months among a nationally representative sample of British children with psychiatric disorder. *Clinical Child Psychology and Psychiatry*. 8, 1, 37-51.

15. Lewis, S., Arseneault L., Caspi, A., et al. (2019). The epidemiology of trauma and post-traumatic stress disorder in a representative cohort of young people in England and Wales. *Lancet Psychiatry*. Vol. 6 (3), 247-256

16. Care Quality Commission (2017). Review of children and young people's mental health services. Phase one report. CQC.

17. Hollis, C., Livingstone, S., & Sonuga-Barke, E. (2020). Editorial: The role of digital technology in children and young people's mental health – a triple-edged sword? *Journal of Child Psychology and Psychiatry*, 61(8), 837-41.

18. National Collaborating Centre for Mental Health (2014). E-therapies systematic review for children and young people with mental health problems. NCCMH, UK

19. Hollis, C., Falconer, C.J., Martin, J.L., et al. (2017). Annual Research Review: Digital health interventions for children and young people with mental health problems – a systematic and meta-review. *Journal of Child Psychology and Psychiatry*, 58(4), 474-503.

20. Christ, C., Schouten, M., Blankers, M., et al. (2020). Internet and computer-based cognitive behavioral therapy for anxiety and depression in adolescents and young adults: Systematic review and meta-analysis. *J Medical Internet Research*. 22, 9, e17831.

For peer review only - http://bmjopen.bmj.com/site/about/guidelines.xhtml

21. National Institute for Clinical Excellence (2019). Depression in children and young people: identification and management. London: NICE.

22. Bevan Jones, R., Stallard, P., Agha, S., et al. (2020). Practitioner review: Co-design of digital mental health technologies with children and young people. *Journal of Child Psychology and Psychiatry*, 61(8), 928-940.

23. Jaycox, L., Ayer, L., Vona, P., et al. (2019). Development and preliminary evaluation of a self-guided, internet-based tool for coping with stress and trauma: Life improvement for teens (LIFT). *Psychological Services*, 16, 1, 85-94

24. Kassam-Adams, N., Marsac, M., Kohser, K., et al. (2016). Pilot Randomized Controlled Trial of a Novel Web-Based Intervention to Prevent Posttraumatic Stress in Children Following Medical Events. *Journal of Pediatric Psychology*. 41(1):138-148.

25. Ruggiero, K., Price, M., Adams, Z., et al. (2015). Web Intervention for Adolescents Affected by Disaster: Population-Based Randomized Controlled Trial. *Journal of the American Academy of Child & Adolescent Psychiatry*. 54(9):709–717.

26. Sijbrandij M, Kunovski I, & Cuijpers P. (2016). Effectiveness of Internet-delivered cognitive behavioral therapy for posttraumatic stress disorder: a systematic review and meta-analysis. *Depression and Anxiety*, 33:783–91.

27. Smith, P., Perrin, S., Yule, W., et al. (2010). *Cognitive therapy for post- traumatic stress disorder*. Routledge Press, London

For peer review only - http://bmjopen.bmj.com/site/about/guidelines.xhtml

28. Ehlers, A., Wild, J., Warnock-Parkes, E., et al. (2020). Randomised controlled trial of therapist-assisted online psychological therapies for post-traumatic stress disorder (STOP-PTSD): trial protocol. *Trials*, 21:355. doi: 10.1186/s13063-020-4176-8

29. Wild, J., Warnock-Parkes, E., Grey, N., et al. (2016). Internet-delivered cognitive therapy for PTSD: a development pilot series, *European J Psychotrauma,* 7: 31019. doi:10.3402/ejpt.v7.310197

30. Hiller, R., Meiser-Stedman, R., Fearon, P., et al. (2016). Research Review: Changes in the prevalence and symptom severity of child post-traumatic stress disorder in the year following trauma – a meta-analytic study. *J Child Psychiat & Psychol*. 57, 8, 884-898.

31. Pynoos, R. S., Weathers, F. W., Steinberg, A. M., et al. (2015). *Clinician-Administered PTSD Scale for DSM-5 - Child/Adolescent Version*

32. Foa, E.B., Asnaani, A., Zang, Y., Capaldi, S., & Yen, R. (2018). Psychometrics of the child PTSD symptom scale (CPSS) for DSM-5 for trauma-exposed children and adolescents. Journal of Clinical Child and Adolescent Psychology, 47, 33-46

33. Dyregrov, A., & Yule, W. (1995). Screening measures: The development of the UNICEF screening battery. In annual meeting of the International Society for Traumatic Stress Studies, Boston, MA.

34. Perrin, S., Meiser-Stedman, R., & Smith, P. (2005). The Children's Revised Impact of Event Scale (CRIES): validity as a screening instrument for PTSD. *Behavioural and Cognitive Psychotherapy*, 33(4), 487-498.

For peer review only - http://bmjopen.bmj.com/site/about/guidelines.xhtml

35. Chorpita, B. F., Yim, L., Moffitt, C., Umemoto, L.A., & Francis, S.E. (2000). Assessment of symptoms of DSM-IV anxiety and depression in children: a revised child anxiety and depression scale. *Behaviour Research & Therapy,* 38, 835-855.

36. Goodman, R. (2001). Psychometric properties of the strengths and difficulties questionnaire. *Journal of the American Academy of Child and Adolescent Psychiatry*, 40 (11), 1337-1345.

37. McKinnon, A., Smith, P., Bryant, R., et al. (2016). An update on the clinical utility of the children's post-traumatic cognitions inventory. *Journal of Traumatic Stress*, 29(3), 253-258.

38. Meiser-Stedman, R., Smith, P., Yule, W. et al. (2007). The Trauma Memory Quality Questionnaire: Preliminary development and validation of a measure of trauma memory characteristics for children and adolescents. *Memory*, 15(3), 271-279.

39. Meiser-Stedman, R., Shepperd, A., Glucksman, E., et al. (2014). Thought control strategies and rumination in youth with acute stress disorder and posttraumatic stress disorder following single-event trauma. *Journal of Child and Adolescent Psychopharmacology*, 24(1), 47–51.

40. Stevens, K. (2012). Valuation of the child health utility 9D index. *Pharmacoeconomics*, 30(8), 729-747.

41. Shearer, J., Papanikolaou, N., Meiser-Stedman, R., et al. (2018). Cost-effectiveness of cognitive therapy as an early intervention for PTSD in children and adolescents: a trial-based evaluation and model. *Journal of Child Psychology and Psychiatry*, 59(7), 773-780.

For peer review only - http://bmjopen.bmj.com/site/about/guidelines.xhtml

42. Eldridge, S. M., Lancaster, G. A., Campbell, M. J., et al. (2016). Defining feasibility and pilot studies in preparation for randomised controlled trials: development of a conceptual framework. *PloS One*, 11(3), e0150205.

43. Thabane, L., Hopewell, S., Lancaster, G.A., et al. (2016). Methods and processes for development of a CONSORT extension for reporting pilot randomized controlled trials. *Pilot Feasibility Studies*, 20;2:25.

44. Vanderweele, T.J., & Vansteelandt, S. (2010). Odds Ratios for Mediation Analysis for a Dichotomous Outcome. *Am J Epidemiol*, 172(12):1339–48.

45. Valeri, L., & Vanderweele, T,J. (2013). Mediation analysis allowing for exposure-mediator interactions and causal interpretation: theoretical assumptions and implementation with SAS and SPSS macros. *Psychol Methods*, 18(2):137–50.

46. Fritz, M. S., Taylor, A. B., & MacKinnon, D. P. (2012). Explanation of two anomalous results in statistical mediation analysis. *Multivariate Behavioral Research*, 47(1), 61-87.

For peer review only

Acknowledgements

We are very grateful to the young people who helped to shape the project and made key contributions to the design of the intervention, and to the young people and carers who participate in the trial.

We are very grateful to the Trial Steering Committee (Cathy Creswell, Andrew Brand, Rachel Calam, & Paul Stallard) for their advice and support.

EC, GF and KG's contributions represent independent research part funded by the NIHR Biomedical Research Centre (South London and Maudsley NHS Foundation Trust and King's College London). KG receives funding from the NIHR Applied Research Collaboration South London (King's College Hospital NHS Foundation Trust). The views expressed are those of the authors and not necessarily those of the NHS, the NIHR, the Department of Health and Social Care.

Figure 1 Study flowchart

For peer review only

Figure 1 Study flowchart

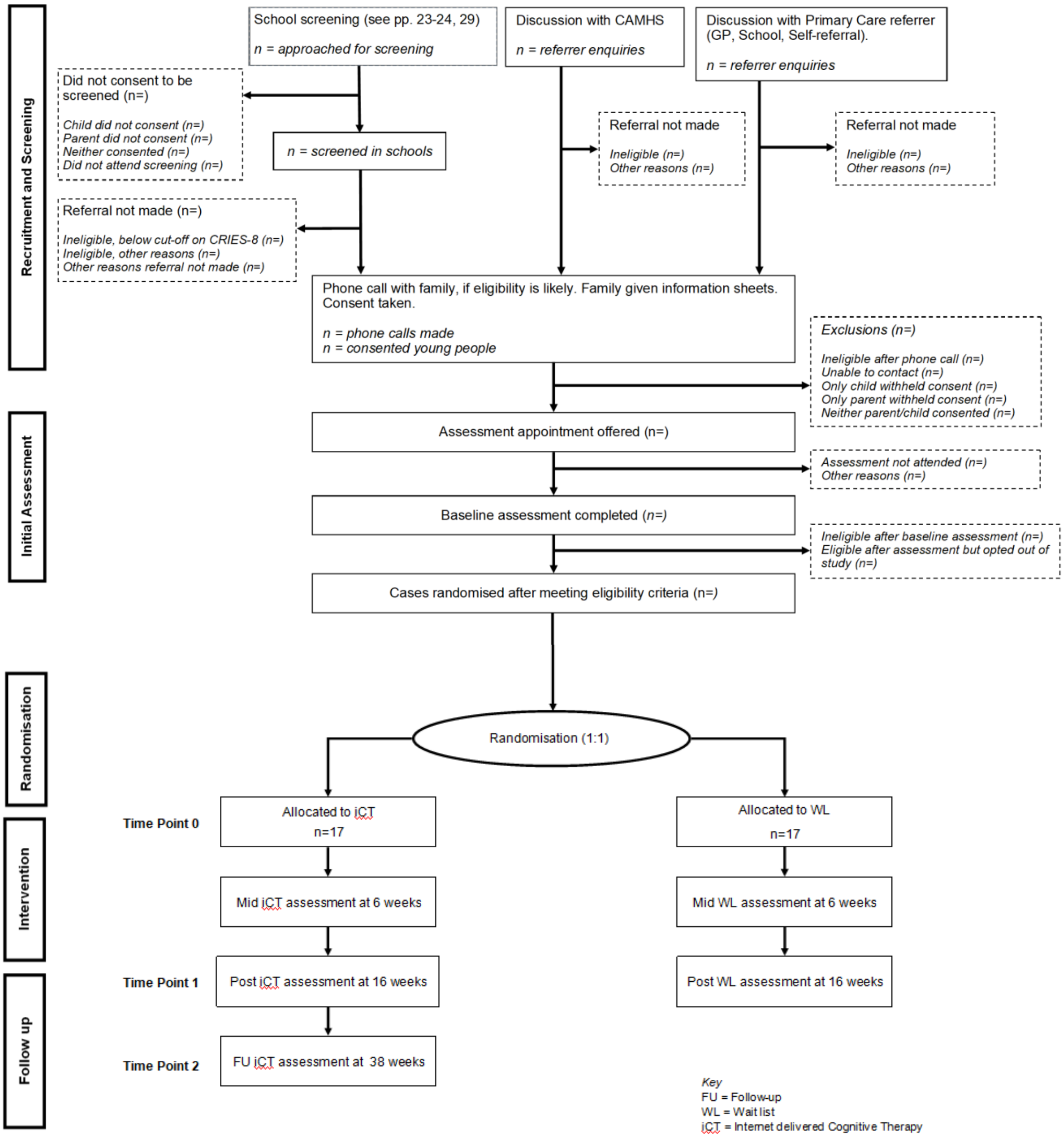

Online PTSD Treatment for Young People & Carers

PARTICIPANT CONSENT FORM

This consent form is for young people aged 16+

Online PTSD treatment for Young People and their Carers (OPTYC): RCT
Dr Patrick Smith

1. I confirm that I have read the information sheet dated 06/05/2020 (version 1.2) for the above study. I have had the opportunity to consider the information, ask questions and have had these answered satisfactorily. ☐

2. I understand that my participation is voluntary and that I am free to withdraw at any time without giving any reason, without my medical care or legal rights being affected. ☐

3. I understand that relevant data collected during the study may be looked at by individuals from King's College London, from regulatory authorities or from the NHS Trust, where it is relevant to my taking part in this research. I give permission for these individuals to have access to my records. ☐

4. I understand that the information collected about me will be used to support other research in the future, and may be shared anonymously with other researchers. ☐

5. I understand that my relevant confidential information will be disclosed to appropriate professionals, including my GP, if a clinical or research worker on the study becomes concerned about my own, or someone else's safety. ☐

6. I agree to my General Practitioner being informed of my participation in the study and being involved in the study, including any necessary exchange of information about me between my GP and the research team. ☐

7. I understand that the information held and maintained by South London and Maudsley NHS Foundation Trust [to be localised] may be used to help contact me or provide information about my health status. ☐

8. I consent to the recording of an interview with me being made and kept on videotape/audiotape. I understand that this recording may be used for purposes of this research project.

9. I agree to take part in the above study. ☐

___________________________	_________________	______________________
Name of Participant	Date	Signature
______________________________	_________________	______________________
Name of Person taking consent	Date	Signature

Online PTSD Treatment for Young People & Carers

PARTICIPANT ASSENT FORM

This assent form is for young people aged 12-15

Please complete this form after you have read the Information Sheet or listened to an explanation about the research.

Online PTSD treatment for Young People and their Carers (OPTYC): RCT
Dr Patrick Smith

1. I confirm that I have read the information sheet dated 06/05/2020 (version 1.2) for the above study. I have had the opportunity to consider the information, ask questions and have had these answered satisfactorily. ☐

2. I understand that my participation is voluntary. If I decide at any time during the research that I no longer wish to take part, I can tell the researchers and pull out and I don't have to give a reason. If I pull out it will not affect my medical care or legal rights. ☐

3. I understand that relevant data collected during the study may be looked at by individuals from King's College London, from regulatory authorities or from the NHS Trust, where it is relevant to my taking part in this research. I give permission for these individuals to have access to my records. ☐

4. I understand that the information collected about me will be used to support other research in the future, and may be shared anonymously with other researchers. ☐

5. I understand that my relevant confidential information will be disclosed to appropriate professionals, including my GP, if a clinical or research worker on the study becomes concerned about my own, or someone else's safety. ☐

6. I agree to my General Practitioner being informed of my participation in the study and being involved in the study, including any necessary exchange of information about me between my GP and the research team. ☐

7. I understand that the information held and maintained by South London and Maudsley NHS Foundation Trust [to be localised] may be used to help contact me or provide information about my health status. ☐

For peer review only - http://bmjopen.bmj.com/site/about/guidelines.xhtml

Online PTSD Treatment for Young People & Carers

8. I agree that an interview with me can be recorded and kept on videotape/audiotape. I understand that this recording may be used for purposes of this research project. ☐

9. I understand that because I am under 16 years old, I can provide my informed assent to take part in this study, but my parent/carer will also need to provide formal consent for me to take part. I agree to take part in the above study. ☐

______________________________	______________	____________________
Name of Participant	Date	Signature

______________________________	______________	____________________
Name of Person taking consent	Date	Signature

Online PTSD Treatment for Young People & Carers

PARENTAL CONSENT FORM

This consent form is for parents/carers of participants aged 12-15

Online PTSD treatment for Young People and their Carers (OPTYC): RCT
Dr Patrick Smith

1. I confirm that I have read the information sheet v1.2 dated 06.05.2020 for the above study. I have been consulted about my child's participation in this research project. I have had the opportunity to ask questions about the study and understand what is involved. I agree to their taking part in this research. ☐

2. I understand that my child's participation is voluntary and that I can request that they are withdrawn from the study at any time without giving any reason, and without their medical care or legal rights being affected. ☐

3. I understand that relevant data collected during the study about my child, may be looked at by individuals from King's College London, from regulatory authorities or from the NHS Trust, where it is relevant to their taking part in this research. I give permission for these individuals to have access to my child's records. ☐

4. I understand that the information collected about my child will be used to support other research in the future, and may be shared anonymously with other researchers. ☐

5. I understand that my child's relevant confidential information will be disclosed to appropriate professionals, including their GP, if a clinical or research worker on the study becomes concerned about my child's, or someone else's safety. ☐

6. I agree to my child's General Practitioner being informed of their participation in the study and being involved in the study, including any necessary exchange of information about them between their GP and the research team. ☐

7. I understand that the information held and maintained by South London and Maudsley NHS Foundation Trust [to be localised] may be used to help contact my child or provide information about their health status. ☐

8. I agree for my child's assessment, and if relevant their treatment sessions, to be audio/video recorded. I understand that this recording may be used for the purposes of this research project. ☐

9. I agree for my child to take part in the above study. ☐

____________________	____________________	____________	________________
Your Name	Relationship to child	Date	Signature

Online PTSD Treatment for Young People & Carers

Name of Person taking consent	Date	Signature

Online PTSD Treatment for Young People & Carers

PARTICIPANT CONSENT FORM FOR PARENTS/CARERS

This consent form is for parents/carers who wish to take part in the study

Online PTSD treatment for Young People and their Carers (OPTYC): RCT
Dr Patrick Smith

1. I confirm that I have read the information sheet dated 06/05/2020 (version 1.2) for the above study. I have had the opportunity to consider the information, ask questions and have had these answered satisfactorily. ☐

2. I understand that my participation is voluntary and that I am free to withdraw at any time without giving any reason, without my medical care or legal rights being affected. ☐

3. I understand that relevant data collected during the study may be looked at by individuals from King's College London, from regulatory authorities or from the NHS Trust, where it is relevant to my taking part in this research. I give permission for these individuals to have access to my records. ☐

4. I understand that the information collected about me will be used to support other research in the future, and may be shared anonymously with other researchers. ☐

5. I understand that the information held and maintained by South London and Maudsley NHS Foundation Trust [to be localised] may be used to help contact me or provide information about my health status. ☐

6. I understand that my relevant confidential information will be disclosed to appropriate professionals, including my GP, if a clinical or research worker on the study becomes concerned about my own, or someone else's safety. ☐

7. I consent to the recording of an interview with me being made and kept on videotape/audiotape. I understand that this recording may be used for purposes of this research project. ☐

8. I agree to take part in the above study. ☐

____________________	____________________	____________________
Name of Participant	Date	Signature
____________________	____________________	____________________
Name of Person taking consent	Date	Signature

Online PTSD Treatment for Young People & Carers

PARTICIPANT CONSENT FORM

This consent form is for young people aged 16+

Online PTSD treatment for Young People and their Carers (OPTYC): School Screening

Dr Patrick Smith

Please initial box

1. I confirm that I have read the information sheet dated 06.05.2020 (version 1.2) for the school screening for the OPTYC study. I have had the opportunity to consider the information, ask questions and have had these answered satisfactorily. ☐

2. I understand that my participation is voluntary and that I am free to withdraw at any time without giving any reason, without my medical care or legal rights being affected. ☐

3. I understand that relevant data collected during the study may be looked at by individuals from King's College London, from regulatory authorities or from the NHS Trust, where it is relevant to my taking part in this research. I give permission for these individuals to have access to my records. ☐

4. I understand that the information collected about me will be used to support other research in the future and may be shared **anonymously** with other researchers. ☐

5. I understand that my relevant confidential information will be disclosed to my parent/carer and appropriate professionals, including my General Practitioner (GP), if a clinical or research worker on the study becomes concerned about my own, or someone else's safety. ☐

6. I agree to my GP being informed of my participation in the study, including any necessary exchange of information about me between my GP and the research team. ☐

7. I agree to take part in the school screening part of this study. ☐

8. I consent to you contacting me via the details provided below. ☐

PLEASE TURN OVER

Online PTSD Treatment for Young People & Carers

Your Name ____

Your School and Form ____

Your Contact Telephone Number ____

Your Home Address ____

Your Email Address ____

Name of Participant	Date	Signature

<u>PLEASE RETURN THIS FORM TO YOUR FORM TUTOR / SCHOOL RECEPTION STAFF / VIA THE PROVIDED FREEPOST ENVELOPE</u>

To be signed by member of OPTYC Team:

Name of Person taking consent	Date	Signature

Online PTSD Treatment for Young People & Carers

don’t have to give a reason. I understand that if I pull out it will not affect my medical

individuals from King’s College London, from regulatory authorities or from the NHS

someone else’s safety.

OPTYC
Online PTSD Treatment for Young People & Carers

King's College London

MRC Medical Research Council

NHS South London and Maudsley NHS Foundation Trust

Online PTSD Treatment for Young People & Carers

PARENTAL CONSENT FORM

This consent form is for parents/carers of participants aged 12-15

Online PTSD treatment for Young People and their Carers (OPTYC): School Screening

Dr Patrick Smith

1. I confirm that I have been consulted about my child's participation in the '**school screening**' part of this research project. I have read the information sheet dated 06.05.2020 (version 1.2) for the school screening and have had the opportunity to ask questions about the study and understand what is involved. ☐

2. I understand that my child's participation is voluntary and that I can request that they are withdrawn from the study at any time without giving any reason, and without their medical care or legal rights being affected. ☐

3. I understand that relevant data collected during the study about my child, may be looked at by individuals from King's College London, from regulatory authorities or from the NHS Trust, where it is relevant to their taking part in this research. I give permission for these individuals to have access to my child's records. ☐

4. I understand that the information collected about my child will be used to support other research in the future and may be shared **anonymously** with other researchers. ☐

5. I understand that my child's relevant confidential information will be disclosed to appropriate professionals, including their General Practitioner (GP), if a clinical or research worker on the study becomes concerned about my child's, or someone else's safety. ☐

6. I agree to my child's GP being informed of their participation in the study, including any necessary exchange of information about them between their GP and the research team. ☐

7. I agree for my child to take part in the school screening part of this study. ☐

8. I consent to you contacting my child and me via the details provided below. ☐

PLEASE TURN OVER

Online PTSD Treatment for Young People & Carers

Your Child's Name ____

Your Child's School and Form ____________________________________

Your Child's Contact Telephone Number ____________________________

Your Child's Email Address ______________________________________

Your Contact Telephone Number __________________________________

Your Home Address ____

Your Email Address ____

____________________	____________________	____________________	
Your Name	Relationship to child	Date	Signature

PLEASE RETURN THIS FORM TO YOUR CHILD'S FORM TUTOR / SCHOOL RECEPTION STAFF/ VIA THE PROVIDED PRE-PAID ENVELOPE

To be signed by member of OPTYC Team:

____________________	____________	______________
Name of Person taking consent	Date	Signature

Checklist: World Health Organization Trial Registration Data Set

Data category	Information
Primary registry and trial identifying number	ISRCTN 16876240
Date of registration in primary registry	06/07/20
Secondary identifying numbers	N/A
Source(s) of monetary or material support	Medical Research Council UK
Primary sponsor	Joint Institute of Psychiatry, Psychology & Neuroscience and the South London and Maudsley NHS Foundation Trust.
Secondary sponsor(s)	N/A
Contact for public queries	Dr Patrick Smith, 020 7848 0506, patrick.smith@kcl.ac.uk
Contact for scientific queries	Dr Patrick Smith, as above
Public title	Online post-traumatic stress disorder treatment for young people and their carers
Scientific title	As above
Countries of recruitment	UK
Health condition(s) or problem(s) studied	Post-Traumatic Stress Disorder (PTSD)
Intervention(s)	Internet delivered Cognitive Therapy for PTSD in Young People (iCT-PTSD-YP)
Key inclusion and exclusion criteria	Young people:<br>1. Aged 12-17 years old<br>2. Main presenting problem is PTSD (diagnosed using CAPS-5-CA) and there is a not a co-morbid problem that would preclude treatment of PTSD<br>3. PTSD symptoms relate to a single trauma<br>4. Participant has access to compatible smartphone or larger computing device (e.g. laptop, desktop computer, iPad) with internet access and to a safe and confidential space in which to engage in iCT<br>5. Participant speaks English to a level that allows therapy without the need for an interpreter, and reads English to a level that allows independent use of iCT<br><br>Parents or carers:<br>1. Parent or carer of a young person who meets all of the inclusion criteria above and none of the exclusion criteria below<br>2. Parent or carer speaks English to a level that allows participation in therapy without the need for an interpreter, and reads English to a level that allows independent use of iCT<br>3. Parent or carer has access to compatible smartphone or larger computing device (e.g. laptop, desktop computer, iPad) with internet access

For peer review only

Study type	Two-arm parallel-group single-blind (outcome assessor) early-stage randomized controlled trial
Date of first enrolment	25/08/20
Target sample size	34
Recruitment status	recruiting
Primary outcome(s)	Feasibility<br>As this is an early-stage trial, the primary outcomes are feasibility outcomes and adherence metrics. Feasibility data on acceptability, compliance, retention, and delivery will be collected.<br><br>Clinical<br>The primary clinical outcome is presence or absence of PTSD 16 weeks after randomisation, determined by administration of a gold standard semi-structured interview by a trained reliable assessor who is blind to treatment allocation.
Key secondary outcomes	Secondary clinical outcomes are continuous scores on a battery of reliable and valid questionnaires measuring severity of PTSD, anxiety, and depression, completed by young people and carers.

For peer review only

Reporting checklist for protocol of a clinical trial.

Based on the SPIRIT guidelines.

Instructions to authors

Complete this checklist by entering the page numbers from your manuscript where readers will find each of the items listed below.

Your article may not currently address all the items on the checklist. Please modify your text to include the missing information. If you are certain that an item does not apply, please write "n/a" and provide a short explanation.

Upload your completed checklist as an extra file when you submit to a journal.

In your methods section, say that you used the SPIRITreporting guidelines, and cite them as:

Chan A-W, Tetzlaff JM, Gøtzsche PC, Altman DG, Mann H, Berlin J, Dickersin K, Hróbjartsson A, Schulz KF, Parulekar WR, Krleža-Jerić K, Laupacis A, Moher D. SPIRIT 2013 Explanation and Elaboration: Guidance for protocols of clinical trials. BMJ. 2013;346:e7586

		Reporting Item	Page Number
**Administrative information**			
Title	#1	Descriptive title identifying the study design, population, interventions, and, if applicable, trial acronym	1
Trial registration	#2a	Trial identifier and registry name. If not yet registered, name of intended registry	3
Trial registration: data set	#2b	All items from the World Health Organization Trial Registration Data Set	3, Appendix 1
Protocol version	#3	Date and version identifier	4
Funding	#4	Sources and types of financial, material, and other support	25
Roles and responsibilities: contributorship	#5a	Names, affiliations, and roles of protocol contributors	1, 25

For peer review only

Roles and responsibilities: sponsor contact information	#5b	Name and contact information for the trial sponsor	5
Roles and responsibilities: sponsor and funder	#5c	Role of study sponsor and funders, if any, in study design; collection, management, analysis, and interpretation of data; writing of the report; and the decision to submit the report for publication, including whether they will have ultimate authority over any of these activities	5
Roles and responsibilities: committees	#5d	Composition, roles, and responsibilities of the coordinating centre, steering committee, endpoint adjudication committee, data management team, and other individuals or groups overseeing the trial, if applicable (see Item 21a for data monitoring committee)	21
**Introduction**			
Background and rationale	#6a	Description of research question and justification for undertaking the trial, including summary of relevant studies (published and unpublished) examining benefits and harms for each intervention	6
Background and rationale: choice of comparators	#6b	Explanation for choice of comparators	11
Objectives	#7	Specific objectives or hypotheses	8
Trial design	#8	Description of trial design including type of trial (eg, parallel group, crossover, factorial, single group), allocation ratio, and framework (eg, superiority, equivalence, non-inferiority, exploratory)	8
**Methods: Participants, interventions, and outcomes**			

For peer review only

Study setting	#9	Description of study settings (eg, community clinic, academic hospital) and list of countries where data will be collected. Reference to where list of study sites can be obtained	9
Eligibility criteria	#10	Inclusion and exclusion criteria for participants. If applicable, eligibility criteria for study centres and individuals who will perform the interventions (eg, surgeons, psychotherapists)	9, 10
Interventions: description	#11a	Interventions for each group with sufficient detail to allow replication, including how and when they will be administered	10-11
Interventions: modifications	#11b	Criteria for discontinuing or modifying allocated interventions for a given trial participant (eg, drug dose change in response to harms, participant request, or improving / worsening disease)	11-12
Interventions: adherence	#11c	Strategies to improve adherence to intervention protocols, and any procedures for monitoring adherence (eg, drug tablet return; laboratory tests)	13
Interventions: concomitant care	#11d	Relevant concomitant care and interventions that are permitted or prohibited during the trial	9
Outcomes	#12	Primary, secondary, and other outcomes, including the specific measurement variable (eg, systolic blood pressure), analysis metric (eg, change from baseline, final value, time to event), method of aggregation (eg, median, proportion), and time point for each outcome. Explanation of the clinical relevance of chosen efficacy and harm outcomes is strongly recommended	12-16<br>table 1
Participant timeline	#13	Time schedule of enrolment, interventions (including any run-ins and washouts), assessments, and visits for participants. A schematic diagram is highly recommended (see Figure)	16

For peer review only

Sample size	#14	Estimated number of participants needed to achieve study objectives and how it was determined, including clinical and statistical assumptions supporting any sample size calculations	16
Recruitment	#15	Strategies for achieving adequate participant enrolment to reach target sample size	16-17
**Methods: Assignment of interventions (for controlled trials)**			
Allocation: sequence generation	#16a	Method of generating the allocation sequence (eg, computer-generated random numbers), and list of any factors for stratification. To reduce predictability of a random sequence, details of any planned restriction (eg, blocking) should be provided in a separate document that is unavailable to those who enrol participants or assign interventions	17
Allocation concealment mechanism	#16b	Mechanism of implementing the allocation sequence (eg, central telephone; sequentially numbered, opaque, sealed envelopes), describing any steps to conceal the sequence until interventions are assigned	17
Allocation: implementation	#16c	Who will generate the allocation sequence, who will enrol participants, and who will assign participants to interventions	17
Blinding (masking)	#17a	Who will be blinded after assignment to interventions (eg, trial participants, care providers, outcome assessors, data analysts), and how	17
Blinding (masking): emergency unblinding	#17b	If blinded, circumstances under which unblinding is permissible, and procedure for revealing a participant's allocated intervention during the trial	n/a

For peer review only

**Methods: Data collection, management, and analysis**			
Data collection plan	#18a	Plans for assessment and collection of outcome, baseline, and other trial data, including any related processes to promote data quality (eg, duplicate measurements, training of assessors) and a description of study instruments (eg, questionnaires, laboratory tests) along with their reliability and validity, if known. Reference to where data collection forms can be found, if not in the protocol	17-18
Data collection plan: retention	#18b	Plans to promote participant retention and complete follow-up, including list of any outcome data to be collected for participants who discontinue or deviate from intervention protocols	12, 19
Data management	#19	Plans for data entry, coding, security, and storage, including any related processes to promote data quality (eg, double data entry; range checks for data values). Reference to where details of data management procedures can be found, if not in the protocol	18
Statistics: outcomes	#20a	Statistical methods for analysing primary and secondary outcomes. Reference to where other details of the statistical analysis plan can be found, if not in the protocol	18
Statistics: additional analyses	#20b	Methods for any additional analyses (eg, subgroup and adjusted analyses)	19 - 22
Statistics: analysis population and missing data	#20c	Definition of analysis population relating to protocol non-adherence (eg, as randomised analysis), and any statistical methods to handle missing data (eg, multiple imputation)	19, 20
**Methods: Monitoring**			

For peer review only

Data monitoring: formal committee	#21a	Composition of data monitoring committee (DMC); summary of its role and reporting structure; statement of whether it is independent from the sponsor and competing interests; and reference to where further details about its charter can be found, if not in the protocol. Alternatively, an explanation of why a DMC is not needed	21
Data monitoring: interim analysis	#21b	Description of any interim analyses and stopping guidelines, including who will have access to these interim results and make the final decision to terminate the trial	19
Harms	#22	Plans for collecting, assessing, reporting, and managing solicited and spontaneously reported adverse events and other unintended effects of trial interventions or trial conduct	22
Auditing	#23	Frequency and procedures for auditing trial conduct, if any, and whether the process will be independent from investigators and the sponsor	n/a
**Ethics and dissemination**			
Research ethics approval	#24	Plans for seeking research ethics committee / institutional review board (REC / IRB) approval	22
Protocol amendments	#25	Plans for communicating important protocol modifications (eg, changes to eligibility criteria, outcomes, analyses) to relevant parties (eg, investigators, REC / IRBs, trial participants, trial registries, journals, regulators)	22
Consent or assent	#26a	Who will obtain informed consent or assent from potential trial participants or authorised surrogates, and how (see Item 32)	23
Consent or assent: ancillary studies	#26b	Additional consent provisions for collection and use of participant data and biological specimens in ancillary studies, if applicable	n/a

For peer review only

Confidentiality	#27	How personal information about potential and enrolled participants will be collected, shared, and maintained in order to protect confidentiality before, during, and after the trial	23
Declaration of interests	#28	Financial and other competing interests for principal investigators for the overall trial and each study site	23
Data access	#29	Statement of who will have access to the final trial dataset, and disclosure of contractual agreements that limit such access for investigators	23-24
Ancillary and post trial care	#30	Provisions, if any, for ancillary and post-trial care, and for compensation to those who suffer harm from trial participation	n/a
Dissemination policy: trial results	#31a	Plans for investigators and sponsor to communicate trial results to participants, healthcare professionals, the public, and other relevant groups (eg, via publication, reporting in results databases, or other data sharing arrangements), including any publication restrictions	24
Dissemination policy: authorship	#31b	Authorship eligibility guidelines and any intended use of professional writers	n/a
Dissemination policy: reproducible research	#31c	Plans, if any, for granting public access to the full protocol, participant-level dataset, and statistical code	24
**Appendices**			
Informed consent materials	#32	Model consent form and other related documentation given to participants and authorised surrogates	Supplementary file uploaded to BMJ site
Biological specimens	#33	Plans for collection, laboratory evaluation, and storage of biological specimens for genetic or molecular analysis in the current trial and for future use in ancillary studies, if applicable	n/a

For peer review only

None The SPIRIT Explanation and Elaboration paper is distributed under the terms of the Creative Commons Attribution License CC-BY-NC. This checklist can be completed online using https://www.goodreports.org/, a tool made by the EQUATOR Network in collaboration with Penelope.ai

For peer review only

BMJ Open

Therapist-supported online cognitive therapy for post-traumatic stress disorder (PTSD) in young people: protocol for an early-stage, parallel-group, randomised controlled study (OPTYC trial)

Journal:	*BMJ Open*
Manuscript ID	bmjopen-2021-054852.R1
Article Type:	Protocol
Date Submitted by the Author:	14-Dec-2021
Complete List of Authors:	Smith, Patrick; King's College London Institute of Psychiatry Psychology and Neuroscience, Psychology<br>Ehlers, Anke; Oxford University, Experimental Psychology<br>Carr, Ewan ; King's College London Institute of Psychiatry Psychology and Neuroscience, Biostatistics and Health Informatics<br>Clark, David; Oxford University, Experimental Psychology<br>Dalgleish, Tim; Cambridge University, Medical Research Council Cognition and Brain Sciences Unit<br>Forbes, Gordon; King's College London Institute of Psychiatry Psychology and Neuroscience, Biostatistics and Health Informatics<br>Goldsmith, Kimberley; King's College London Institute of Psychiatry Psychology and Neuroscience, Biostatistics and Health Informatics<br>Griffiths, Helena; King's College London Institute of Psychiatry Psychology and Neuroscience, Psychology<br>Gupta, Monica; King's College London Institute of Psychiatry Psychology and Neuroscience, Psychology<br>King, Dorothy; King's College London Institute of Psychiatry Psychology and Neuroscience, Psychology<br>Miles, Sarah; King's College London Institute of Psychiatry Psychology and Neuroscience, Psychology<br>Plant, Dominic; King's College London Institute of Psychiatry Psychology and Neuroscience, Psychology<br>Yule, William; King's College London Institute of Psychiatry Psychology and Neuroscience, Psychology<br>Meiser-Stedman , Richard ; University of East Anglia, Department of Clinical Psychology & Psychological Therapies
**Primary Subject Heading**:	Mental health
Secondary Subject Heading:	Mental health
Keywords:	MENTAL HEALTH, Child & adolescent psychiatry < PSYCHIATRY, Anxiety disorders < PSYCHIATRY

Therapist-supported online cognitive therapy for post-traumatic stress disorder (PTSD) in young people: protocol for an early-stage, parallel-group, randomised controlled study (OPTYC trial)

Patrick Smith[1,2], Anke Ehlers[3], Ewan Carr[4], David M Clark[3], Tim Dalgleish[5,6], Gordon Forbes[4], Kim Goldsmith[4], Helena Griffiths[1], Monica Gupta[1], Dorothy King[1], Sarah Miles[1 2], Dominic T Plant[1], William Yule[1], Richard Meiser-Stedman[7]

1. Department of Psychology, Institute of Psychiatry, Psychology and Neuroscience, King's College London
2. South London & Maudsley NHS Foundation Trust
3. Department of Experimental Psychology, University of Oxford
4. Department of Biostatistics and Health Informatics, Institute of Psychiatry, Psychology and Neuroscience, King's College London
5. Medical Research Council Cognition and Brain Sciences Unit, University of Cambridge
6. Cambridgeshire and Peterborough NHS Foundation Trust
7. Department of Clinical Psychology & Psychological Therapies, Norwich Medical School, University of East Anglia

Corresponding author:

Patrick Smith, Department of Psychology, Institute of Psychiatry, Psychology and Neuroscience, King's College London, London SE5 8AF, email patrick.smith@kcl.ac.uk

Word count

4738 (excluding title page, abstract, references, figures, tables & acknowledgments)

For peer review only

For peer review only - http://bmjopen.bmj.com/site/about/guidelines.xhtml

ABSTRACT

Introduction

Post-Traumatic Stress Disorder (PTSD) is a disabling psychiatric condition that affects a significant minority of young people exposed to traumatic events. Effective face-to-face psychological treatments for PTSD exist. However, most young people with PTSD do not receive evidence-based treatment. Remotely delivered digital interventions, have potential to significantly improve treatment accessibility. Digital interventions have been successfully employed for young people with depression and anxiety, and for adults with PTSD. However, digital interventions to treat PTSD in young people have not been evaluated. The Online PTSD Treatment for Young People & Carers (OPTYC) trial will evaluate the feasibility, acceptability, and initial indications of clinical efficacy of a novel internet-delivered Cognitive Therapy for treatment of PTSD in young people (iCT-PTSD-YP).

Methods and analysis

This protocol describes a two-arm, parallel-groups, single-blind (outcome assessor), early-stage RCT, comparing iCT-PTSD-YP with a Waiting List (WL) comparator. N=34 adolescents (12-17 years old), whose primary problem is PTSD after exposure to a single traumatic event, will be recruited from 14 NHS Child and Adolescent Mental Health Services (CAMHS) in London and southeast England, from secondary schools and primary care in the same region, or via self-referral from anywhere in the UK using the study website. Individual patient-level randomization will allocate participants in a 1:1 ratio, randomised using minimisation according to sex and baseline symptom severity. The primary study outcomes are data on feasibility and acceptability, including recruitment, adherence, retention, and adverse events. . The primary clinical outcome is PTSD diagnosis 16-weeks post-randomisation. Secondary clinical outcomes include continuous measures of PTSD, anxiety, and depression symptoms. Regression analyses will provide preliminary estimates of the effect of iCT-

For peer review only - http://bmjopen.bmj.com/site/about/guidelines.xhtml

PTSD-YP on PTSD diagnosis, symptoms of PTSD, anxiety and depression relative to WL. Process-outcome evaluation will consider which mechanisms mediate recovery. Qualitative interviews with young people, families and therapists will evaluate acceptability.

Ethics and dissemination

The study was approved by a UK Health Research Authority (HRA) Research Ethics Committee (REC; 19/LO/1354). For participants aged under 16, informed consent will be provided by carers and the young person will be asked for their assent; participants aged 16 years or older can provide informed consent without their parent or caregiver's involvement. Findings will be disseminated broadly to participants, healthcare professionals, the public, and other relevant groups. Study findings will be published in peer-reviewed journals.

Trial registration

Prospectively registered on 6 July 2020: ISRCTN 16876240

All items from the World Health Organization Trial Registration Data Set are detailed in Appendix 1

For peer review only

For peer review only - http://bmjopen.bmj.com/site/about/guidelines.xhtml

Strengths and limitations of this study

- An early-stage trial to gather data on feasibility, acceptability and initial indications of clinical efficacy of internet delivered Cognitive Therapy for PTSD in young people (iCT-PTSD-YP)
- Young people were extensively involved in designing the phone App and website
- CT-PTSD is theory-based and has demonstrated efficacy when delivered face-to-face and iCT-PTSD is effective in adults
- This trial can be delivered entirely remotely
- This early stage RCT is not powered to detect between group effects

Keywords

PTSD, young people, cognitive therapy, trial, digital mental health

Administrative information

Title

The OPTYC trial: Study protocol for an early stage randomised controlled trial of therapist-supported online cognitive therapy for post-traumatic stress disorder (PTSD) in young people

Registration

Prospectively registered: ISRCTN 16876240

Protocol

Protocol version 1.5 (April 2021)

For peer review only - http://bmjopen.bmj.com/site/about/guidelines.xhtml

Sponsor

Institute of Psychiatry, Psychology & Neuroscience (King's College London) and the South London and Maudsley NHS Foundation Trust. The funder and sponsor approved the study design and capacity to implement. Neither the funder nor the sponsor has a role in collection, management, analysis, or interpretation of data; writing of the report; or decision to submit the report for publication. Neither the funder nor the sponsor has ultimate authority over any of these activities.

For peer review only

INTRODUCTION

Background and rationale

Trauma exposure and Post-Traumatic Stress Disorder (PTSD) are both prevalent among youth under 18 years old. Between 15-82% of youth are exposed to traumas, and between 3-8% of youth will develop PTSD by the age of 18 years [1, 2, 3], representing a significant level of morbidity for health services. For affected individuals, PTSD is highly distressing, causes marked impairments in functioning and may run a chronic course for years or decades if left untreated [4, 5].

Effective treatments for PTSD exist. Recent reviews of psychological treatments for PTSD in youth find that various forms of Trauma-Focused Cognitive Behavioural Therapy (TF-CBT) show consistently large effects in reducing PTSD symptoms and associated comorbidities [6, 7]. Cognitive Therapy for PTSD (CT-PTSD) is a form of TF-CBT developed by our group [8, 9] recommended as a first line intervention in national and international practice guidelines[10]. The treatment is theory-based, manualised, and delivered over 10-12 individual sessions. Two published RCTs [11, 12] find that CT-PTSD is acceptable to young people (8-18 years old), and efficacious [13].

However, most young people under 18 years old with PTSD do not receive an effective, evidence-based treatment. The gap between community prevalence of psychiatric disorders and treatment provision for young people is well-known and longstanding [14]. In a recent population based British study, only 40% of young people with PTSD sought help from GPs or mental health practitioners and only 20% had accessed specialist mental health services in the past year [15]. Limited access to treatment may be due to multiple interacting factors including under-capacity and long waiting times for assessment and treatment in specialist Child and Adolescent Mental Health Services [16], and the burden and inconvenience to young people and families in attending face-to-face appointments in a clinic.

For peer review only - http://bmjopen.bmj.com/site/about/guidelines.xhtml

Remote delivery of psychological therapy via the internet has enormous potential to address some of these barriers, and to increase accessibility of treatment [17]. Young people have enthusiastically endorsed the potential for digital health interventions [18]. For disorders other than PTSD, digital health interventions are known to be acceptable to young people and clinically helpful. For example, Computerised Cognitive Behavioural Therapy (C-CBT) for depression demonstrates clear clinical benefit for young people [19, 20] and is now recommended by NICE [21]. Lessons have been learned about the development of digital mental health interventions including the need for: co-design with young people [22]; and the active engagement of young people in therapy facilitated by continued therapist support during treatment[19].

Development of remotely delivered therapy for treatment of PTSD in young people lags behind that for other disorders. Jaycox and colleagues[23] report encouraging preliminary outcomes for a self-help web-based tool to augment and enhance usual school support services for trauma-exposed youth (7th – 12th Grade, mean age 15 years). Kasam-Adams and colleagues[24] showed that a digital intervention for preventing PTSD symptoms in injured children (8-12 years old) was feasible and clinically promising. Ruggerio and colleagues[25] found that use of a web-based psycho-education intervention for disaster-affected adolescents (mean age 14.5 years) was associated with improvements in PTSD symptoms. However, to our knowledge, no studies have yet reported on the development or evaluation of internet-delivered TF-CBT for treatment of PTSD in children and young people. This is surprising because face to face TF-CBT is well established as an effective treatment for PTSD in youth, and work with adults shows that PTSD is a disorder which is treatable via the internet [26].

In this project we aim to address this clear gap. We have co-designed with adolescents an internet version of CT-PTSD, to be delivered via smartphone App and website, with remote therapist support. Our longer-term intention is to determine whether this approach will help to reduce the treatment gap for young people with PTSD by making an efficacious therapy more widely available.

For peer review only - http://bmjopen.bmj.com/site/about/guidelines.xhtml

Our aim in the current early-stage trial is to gather preliminary data on feasibility, acceptability, and initial signal of clinical effects of internet-delivered Cognitive Therapy for PTSD for young people (iCT-PTSD-YP), relative to a Waiting List (WL) condition. Data gathered in the current trial will be used to inform the design and size of a future scaled-up trial.

Objectives

The primary objective is to provide data on feasibility, acceptability, compliance, retention, and delivery of iCT-PTSD-YP. The secondary objective is to provide initial estimates of the effect of iCT-PTSD-YP on symptoms of PTSD, anxiety and depression relative to a WL condition.

METHODS AND ANALYSIS

Trial design

This study is a two-arm, parallel groups, single-blind (outcome assessor), early stage RCT, comparing iCT-PTSD-YP with a WL comparator. Individual patient-level randomization will allocate participants in a 1:1 ratio, randomised using minimisation according to sex and baseline symptom severity.

Patient and Public Involvement

Members of the BRC Young Person's Mental Health Advisory Group (YPMHAG; 16–25 year-olds with lived experience of using mental health services) were consulted before grant submission: they provided verbal and written feedback on the research ideas. Young people (N=33, aged 12-17 years old) were consulted at an early stage about the design of the App via a series of four focus groups held in four different schools. Young people receiving face-to-face CT-PTSD provided

For peer review only - http://bmjopen.bmj.com/site/about/guidelines.xhtml

feedback on initial prototypes of the App. A young person with lived experience of using mental health services is a member of the Trial Steering Committee (TSC). We will consult the YPMHAG and the TSC about our dissemination strategy.

Study setting

The trial will be carried out in the UK. Trial randomisation will be carried out by King's College London Clinical Trials Unit (CTU). Trial therapists will be based at King's College London and the University of East Anglia. Referrals will be sought from 14 NHS Child and Adolescent Mental Health Services (CAMHS) in London and southeast England, all of which are registered as study sites. Referrals will also be sought from secondary schools and primary care in the same region. We will offer to carry out screening surveys in schools to identify potentially eligible young people (12- 17 years old). Self-referral from anywhere in the UK is also possible via the study website.

Eligibility criteria

Young people are eligible to be included if: they are aged 12-17 years old; their main presenting problem is PTSD and there is a not a co-morbid problem that would preclude treatment of PTSD; PTSD symptoms relate to a single trauma; they speak English to a level that allows therapy without the need for an interpreter, and they read English to a level that allows independent use of iCT; they have access to a smartphone and a larger device (laptop, desktop computer, tablet) with internet access, and they have access to a safe and confidential space in which to engage in iCT. Young people are excluded if they have: brain damage; intellectual disability; Pervasive developmental disorder or neurodevelopmental disorder, as assessed by clinical interview with parents / carers; other psychiatric diagnosis that requires treatment before PTSD, determined by clinical interview and questionnaires; moderate to high risk to self; ongoing trauma-related threat;

For peer review only - http://bmjopen.bmj.com/site/about/guidelines.xhtml

have started treatment with psychotropic medication, or changed medication, within the last 2 months; or are currently receiving another psychological treatment, as assessed in clinical interview; or previously received Trauma-Focused CBT in relation to the same traumatic event that they are currently seeking treatment for.

Parents or carers are eligible to be included if they: are the parent or carer of a young person who meets all of the inclusion criteria and none of the exclusion criteria above; speak English to a level that allows participation in therapy without the need for an interpreter, and read English to a level that allows independent use of iCT; and have access to a smartphone and/or larger device with internet access.

Interventions

iCT-PTSD-YP

Internet-delivered Cognitive Therapy for PTSD for young people (iCT-PTSD-YP) comprises therapist-supported online delivery of all components from our published manual of face-to-face CT-PTSD for young people [27]. Treatment aims to change problematic appraisals, update trauma memories, and change unhelpful coping responses. Treatment components are delivered in modules. There are 10 core modules for all young people (Psychoeducation about PTSD, Reclaiming life, Understanding PTSD, Developing a trauma narrative, Identifying hotspots, Updating the narrative, Working with triggers, Overcoming sense of danger, Visiting the site virtually and/or in person, Developing a blueprint) that are released to the young person sequentially by the therapist, and 11 optional modules which are released according to individual need (Relaxation, Sleep, Working with images, Working with physical difference, Anger, Grief, Shame, Guilt, Self-criticism, Rumination, and Panic). Modules were co-designed with input from young people and built on the content of the modules developed for iCT-PTSD for adults [28, 29]. Modules are interactive (prompting

For peer review only - http://bmjopen.bmj.com/site/about/guidelines.xhtml

for user action to progress through the App and requesting user text input and questionnaire responses) and include text, illustrations, audio case examples, animations, and videos. Modules are intended for independent self-study by young people. Therapists can log onto the site to view young people's progress including their text input and questionnaire responses. Young people and therapists can message each other via the App. Parents and carers are provided a separate log on to the carer version of the App. The carer version comprises 8 modules, and the emphasis is on providing information to carers about therapy, including advice about how carers can help in young people's recovery. Carers do not have access to any information that their child inputs to the App. Modules are delivered via a progressive web App (PWA) on a smartphone or computer, hosted on a secure server. The App is not publicly available currently. For trial participants, an individual account requiring two-factor authentication log-in is created for the young person and their carer.

Therapists will be clinical psychologists or CBT therapists who have received training in face-to-face CT-PTSD, and in use of the iCT-PTSD-YP App. Therapists will have contact with young people and carers via phone or videoconferencing at least once a week for the duration of therapy. Therapists release modules according to the young person's individual formulation, remind and encourage young people to log on to the App, and provide and support in using the App and implementing the treatment components. Weekly clinical supervision will be provided by a consultant clinical psychologist from the trial team.

Therapy is delivered over 12 weeks. Post treatment assessment is carried out one month after the end of treatment (i.e. at 16 weeks after randomisation).

Waiting List

Young people will be placed on a Waiting List (WL) and re-assessed 16 weeks after randomisation. Young people who require treatment at the end of the waiting period will be offered

For peer review only - http://bmjopen.bmj.com/site/about/guidelines.xhtml

immediate iCT-PTSD-YP. WL control arms are commonly used in PTSD treatment trials[6] because natural recovery from PTSD can be substantial [30]. Use of a WL condition ensures that the effect of treatment is not overestimated, and shows whether treatment is impeding the rate of natural recovery.

Withdrawals

Participants will be withdrawn from treatment if: a current illness prevents further treatment; there is a change in the participant's condition or circumstances that in the clinician's opinion justifies the discontinuation of treatment; or the participant withdraws consent for treatment. Participants who discontinue treatment for the above reasons will be invited to provide follow-up data and will remain in the trial for the purposes of data analysis. If the participant no longer wishes to be followed up to provide research data, the participant will be withdrawn entirely from the trial. The different types of withdrawal will be captured and reported.

Outcomes

The schedule for assessments is presented in Table 1.

The primary outcomes for the study are data on feasibility, adherence, and acceptability, which will be reported using the metrics specified below.

Feasibility outcomes

We will report: (1) the number of young people referred to the trial in total and according to referral route; (2) the number of young people screened in schools, and the proportion of those who proceed to a phone call with the family; (3) the number and proportion of young people in schools scoring above cut-off on a validated screening questionnaire (CRIES-8, see below) relative to the

For peer review only - http://bmjopen.bmj.com/site/about/guidelines.xhtml

number of young people screened in schools; (4) the number and proportion of young people in schools who score above cut-off on the screening questionnaire but decline further participation with the trial relative to those scoring above cut-off); (5) the number and proportion of young people in schools who score above cut-off on the screening and consent to further assessment but are deemed ineligible at baseline assessment relative to those deemed eligible at baseline assessment; (6) the number of assessment appointments offered to participants; (7) the number and proportion of assessment appointments attended by participants, relative to the number of appointments offered, reported by referral source; (8) reasons for not attending assessment appointments, reported by referral source; (9) the number and proportion of young people who at baseline assessment consent to participate in the trial, relative to the number who attend assessment, with reasons for not consenting if known; (10) the number and proportion of young people eligible for the trial after baseline assessment, relative to the number of baseline assessments completed; (11) the number and proportion of young people who are randomised, and the proportion of consented young people who are randomised relative to the number who consented; (12) reasons for withdrawing from the trial if known; and (13) the number retained in study at 16 weeks (post-treatment) and at 38 weeks (follow-up), and the proportions of those who start treatment who are retained.

For peer review only

Adherence metrics

For participants allocated to iCT-PTSD-YP, we will report: (1) the number of times logged into the programme per week and in total; (2) time spent logged in per week and in total; (3) the number of modules completed in total and according to device used; (4) the number of therapist phone calls attended per week and in total, and the number of missed phone appointments; (5) time spent on phone calls per week and in total; (6) the number of messages to / from therapist per week and in

For peer review only - http://bmjopen.bmj.com/site/about/guidelines.xhtml

total; (7) the number and proportion of young people who start treatment; (8) the number of weeks

MEASURE	STUDY PERIOD					
	**Screen** 0-1 weeks	**Pre** 0 weeks	**Weekly** (*iCT only*)	**Mid** 0+ 6 weeks	**Post** 0+ 16 weeks	**Follow-up** 0+38 weeks (*iCT only*)
ENROLMENT						
Eligibility screen	x					
Provide study information	*x*					
Gain informed consent		*x*				
ONLINE ASSESSMENT						
DAWBA		*x*				
INTERVIEW						
DEMOGRPAHIC INTERVIEW		*x*				
CAPS-CA-5		*x*			*x*	
CGAS		*x*			*x*	
ADOLESCENT QUESTIONNAIRES						
CPSS-5		*x*			*x*	*x*
CRIES-8		*x*	*x*	*x*	*x*	*x*
RCADS-C		*x*			*x*	*x*
CPTCI		*x*		*x*	*x*	*x*

of therapy completed and (9) reasons for dropping out of treatment if known.

Acceptability outcomes

We will carry out qualitative interviews with young people, carers, and therapists to gauge acceptability of iCT-PTSD-YP, and we will summarise interview data using content analysis. We will aim for these interviews to be representative of individuals involved in the feasibility trial (young people, carers, therapists), including young people who left the study or failed to adhere to the course of treatment, to provide a full range of views. We will interview trial participants in both arms about the acceptability of the research procedures including the assessment measures and their views on randomisation.

For peer review only

For peer review only - http://bmjopen.bmj.com/site/about/guidelines.xhtml

TMQQ	*x*	*x*	*x*	*x*
Rumination items	*x*	*x*	*x*	*x*
CHU-9D	*x*		*x*	*x*
Adverse events		*x*	*x*	*x*
CARER QUESTIONNAIRES				
SDQ-P	*x*		*x*	*x*
RCADS-P	*x*		*x*	*x*
CA-SUS	*x*		*x*	*x*
Adverse events		*x*	*x*	*x*
QUALITATIVE INTERVIEWS				
Adolescents			*x*	
Carers			*x*	
Therapists			*x*	

Table 1 Study schedule

Primary clinical outcome

Presence of PTSD according to the DSM-5 at 16 weeks post-randomisation, ascertained using the Clinician Administered PTSD Scale for DSM-5: Child and Adolescent version (CAPS-CA-5 [30]), administered by trained reliable raters, blind to treatment allocation.

Secondary clinical outcomes

Child-reported outcomes at 16 weeks post randomisation: PTSD symptom severity (continuous score) on the CAPS-CA-5 [31]; PTSD symptom severity on the Child PTSD Symptom Scale for DSM-5 (CPSS-5[32]); PTSD symptom severity on the Children's Revised Impact of Event Scale, 8-item version (CRIES-8 [33, 34]); and symptoms of depression and anxiety on the 25-item Revised Children's Anxiety and Depression Scale (RCADS[35]). Carer reported outcomes at 16 weeks post randomisation: Revised Children's Anxiety and Depression Scale – Parent version (RCADS-P [35]); and Strength & Difficulties Questionnaire – parent version (SDQ-P [36]). At 38-week follow-up for

For peer review only

For peer review only - http://bmjopen.bmj.com/site/about/guidelines.xhtml

participants in the iCT-PTSD-YP only, all secondary clinical outcomes apart from the CAPS-CA-5 will be repeated.

Process measures

The cognitive model [8] on which treatment is based specifies a number of mechanisms of therapeutic change. We will test mediation via changes in appraisals, memory quality, and ruminative thinking from baseline to mid-treatment (6-weeks post randomisation) using: the Child Post Traumatic Cognitions Inventory (CPTCI [37]); the Trauma Memory Quality Questionnaire (TMQQ[38]); and the Trauma Related Rumination Questionnaire items [39].

Health economic outcomes

We will collect economic data on health utilities and resource use using the Child Health Utility Index 9D (CHU-9D [40]) and the Child & Adolescent Service Use Schedule (CA-SUS [41]), administered at baseline and 16-weeks post-randomisation.

Participant timeline

All participants will be assessed three times during the study: pre-treatment (week 0), mid-treatment (week 6 post-randomisation), and post-treatment (week 16 post-randomisation). Participants in iCT-PTSD-YP will complete a brief weekly measure of PTSD symptoms (CRIES-8) and mood (Likert scale) on the App, and a follow up assessment (week 38 post randomisation). The first participant was randomised on 24.08.20, and the last participant was randomised on 20/10.21. The trial is currently closed to new recruitment.

For peer review only - http://bmjopen.bmj.com/site/about/guidelines.xhtml

Sample size

We will recruit 17 participants per arm. In our previous RCTs of face-to-face CT-PTSD [11, 12] in young people, we had 4% drop-out, but we have conservatively allowed for approximately 20% drop-out, to give at least n = 14 at post-treatment in each arm. An early-stage trial of this size will be sufficient to gather meaningful feasibility data on acceptability, compliance, retention, and delivery. Power calculations are not typically used to determine sample size for feasibility studies. Therefore, we acknowledge an insufficient sample size to allow definitive between-group comparisons in this early stage RCT [42, 43].

Recruitment

Participants will be recruited via three routes (see Figure 1): (1) from school screening; (2) from NHS CAMHS teams; and (3) from primary care (GP or school referral) or self-referral. For all referral routes, consent will be sought before assessment, and eligibility will be determined by the clinical assessment.

Allocation

Once a participant is confirmed as eligible and consenting to the study, they will be registered in the main participant database (held using the IBM-SPSS programme). Participants will be randomised to receive iCT-PTSD-YP or WL at a 1:1 ratio. Randomisation will be carried out by the King's Clinical Trials Unit (KCTU) via a web-based service utilising minimisation with a random component. Minimisation factors will be sex and baseline PTSD symptom severity assessed by the CPSS (low: <51, high: ≥51). These factors were chosen in order to balance factors that may affect treatment response across the two arms. Other factors (such as age and trauma type) were not included due to the modest trial size.

For peer review only - http://bmjopen.bmj.com/site/about/guidelines.xhtml

Blinding

All assessors of the primary and secondary clinical outcomes at follow-up at 16 weeks will be blind to trial arm allocation. Blind outcome assessors will be independent research assistants or clinical psychologists who are not part of the trial team. Assessors will be trained to standard on the CAPS-CA-5 interview, and inter-rater reliability will be assessed for 20 randomly selected interviews. The senior trial statistician (KG) will also be blind with all other members of the study team unblind to trial arm allocation. Unblinding of the senior trial statistician and the analysis of outcomes by intervention arm will occur after the initial draft of the statistical analysis report is generated.

Data collection methods

For the primary clinical outcome, the CAPS-CA clinical interview is completed on the phone or via videoconference, with symptom level responses marked on the interview form and then entered into the trial database. For secondary clinical outcomes, questionnaires are completed online via a secure commercial system (Qualtrics) with responses downloaded to an electronic database and re-entered into the trial database. Feasibility outcomes are recorded by the study research assistant in the trial database. Adherence metrics are either recorded by the trial therapist in the study database or automatically captured by the App and downloaded to standard database software.

Data management

Participant Information will be kept confidential and managed in accordance with the Data Protection Act, GDPR policies, NHS Caldicott Guardian, The UK Policy Framework for Health and Social Care Research, and Research Ethics Committee Approval. Personally identifiable data will be

For peer review only - http://bmjopen.bmj.com/site/about/guidelines.xhtml

collected from participants including name and contact details. This information will be stored securely and separately from all other study-generated data, which will be anonymised. Each participant will be given a unique Participant Identification Number (PIN). All feasibility and clinical outcomes for the RCT will be stored in SPSS databases against the participant PIN. These databases will be stored on a secure KCL network drive, accessible to the study team only. Databases will be stored in a version control system, such that changes made over time can be examined and recovered. All databases will be registered in the King's Data Protection Register (KDPR).

Statistical methods

A comprehensive statistical analysis plan (SAP) will be developed and agreed with the Trial Steering Committee (TSC) before any analysis is carried out. The SAP will describe statistical procedures in detail. Quantitative analyses will employ up-to-date versions of statistical software (e.g Stata or R).

Analysis of feasibility outcomes and adherence metrics

The feasibility outcomes and adherence metrics will be summarised with appropriate summary statistics (e.g. means and standard deviations/medians and interquartile ranges for continuous outcomes; frequencies and proportions for count outcomes). Where appropriate some feasibility outcomes will either be reported only for the iCT-PTSD-YP arm or will be reported separately by arm.

Clinical outcomes

For peer review only - http://bmjopen.bmj.com/site/about/guidelines.xhtml

As this is an early-stage trial designed to gather data on feasibility outcomes, it is not powered to detect between-arm differences: where between-arm differences are presented, they will be treated as exploratory and not treated as inferential. Data completeness will be summarised for clinical outcomes. All comparative analyses will primarily be conducted under the intention-to-treat (ITT) principle – all participants with a completed outcome will be included in the analysis and analysed according to the arm they were randomised to. Where deviations from ITT occur, this will be reported. We will carry out per-protocol analyses in addition to ITT, but these analyses will be treated as secondary to the ITT analysis. There will be no interim or subgroup analyses.

The primary and secondary clinical outcomes will be summarised with appropriate summary statistics by trial arm at each time point (primary, frequencies and proportions; secondary, means and standard deviations). For each outcome we will estimate the treatment effect at 16 weeks, with the appropriate 95% confidence interval. The iCT-PTSD-YP versus WL odds ratio for remission from PTSD caseness at 16 weeks post-randomisation will be assessed using logistic regression with trial arm and the minimisation variables as covariates. The iCT-PTSD-YP versus WL mean differences in secondary clinical outcomes at 16 weeks post-randomisation will be estimated using linear regression, with trial arm, baseline outcome score and minimisation variables as covariates.

We will carry out per-protocol analyses for the primary outcome, and the CPSS-5 and CRIES-8 secondary outcomes at 16 weeks. These will be treated as secondary to the ITT analysis. The per protocol analyses will be conducted in two populations. The first will consist of all participants with recorded outcome data who complete the minimum therapy needed to achieve clinical benefit (defined as completing at least the first six core modules (Psychoeducation about PTSD, Reclaiming life, Understanding PTSD, Developing a trauma narrative, Identifying hotspots, Updating the narrative)). The second per protocol population will consist of all participants from the first per protocol population who have additionally completed the core module, "Working with triggers".

For peer review only - http://bmjopen.bmj.com/site/about/guidelines.xhtml

Process outcomes

An exploratory mediation analysis will be carried out to assess the indirect effect of treatment allocation on the primary clinical endpoint via the Child Post Traumatic Cognitions Inventory score (CPTCI), the Trauma Memory Questionnaire (TMQQ), and items relating to rumination, measured at 6 weeks post-randomisation. The total, direct, and indirect effects of treatment allocation on 16-week PTSD caseness will be estimated using the Stata paramed command[44, 45] to properly calculate effects for a binary outcome, along with associated 95% confidence intervals. Confidence intervals for the indirect effect will be estimated using the percentile bootstrap[46].

Health economics

To gauge the feasibility of collecting health economic data, data completeness will be summarised by presenting the number and proportion of complete and missing values at each time point. Efficacy will be measured using the CHU-9D measure of health-related quality of life. Data on iCT-PTSD-YP, contact time and indirect time for the intervention will be collected directly from clinicians and service records. Service use estimates will be combined with standard UK sources for unit costs to estimate total costs. The cost of iCT-PTSD-YP will be directly calculated. These data will allow us to index service use and permit preliminary estimates of the potential cost-effectiveness of iCT-PTSD-YP.

Qualitative analysis

We will carry out qualitative interviews at the end of each participant's iCT-PTSD-YP. If participants drop out of treatment early, we will endeavour to interview them. Semi-structured interviews using a topic guide will be carried out by a member of the study team who was not

For peer review only - http://bmjopen.bmj.com/site/about/guidelines.xhtml

involved in treatment. The views and experiences of patients, parents or carers, and trial clinicians will be sought in order to gain a multi-perspective view of acceptability. Content analysis will be used to explore both commonalities and variations within and between these respondents. We will interview trial participants in both arms about the acceptability of the research procedures including the assessment measures and their views on randomisation. We will invite all participants to take part in qualitative interviews, until data saturation is reached.

Data monitoring

Project oversight will be provided by a monthly Project Management Group (PMG) attended by all co-investigators. Trial oversight will be provided by a 6-monthly Trial Steering Committee (TSC). The TSC will review the protocol, agree the statistical analysis plan (SAP), and safeguard the interests of trial participants. The TSC will provide advice to the CI and sponsor. A separate Data Monitoring Committee (DMC) will not be convened. The TSC will monitor adverse events and adverse reactions and will convene an emergency DMC if needed.

Adverse events

Adverse events (AEs) are defined as any untoward occurrence in a trial participant, including events that are not necessarily caused by or related to trial procedures. Serious adverse events are defined as AEs that result in death, are life-threatening, require hospitalisation or prolong existing hospitalisation, or result in persistent or significant disability or incapacity. Some adverse events are expected in this study, and will be reported to the TSC, for example: self-harm not requiring medical attention, increase in suicidal ideation, worsening of PTSD symptoms (defined as 7-point increase in CRIES-8). Serious AEs will be reported to the Chair of the TSC, the REC, and the sponsor. Adverse events will be assessed at each assessment time point. Risk monitoring including adverse event

For peer review only - http://bmjopen.bmj.com/site/about/guidelines.xhtml

monitoring will be carried out during clinical contact for those allocated to iCT-PTSD-YP. AEs will be monitored and recorded from randomisation to final follow-up.

ETHICS AND DISSEMINATION

Ethical approval

The study was approved by a UK Health Research Authority (HRA) Research Ethics Committee (REC; 19/LO/1354). The study is sponsored by King's College London.

Protocol amendments

We were initially funded to run a 3-arm feasibility RCT comparing iCT-PTSD-YP with face-to-face CT-PTSD and WL. The COVID-19 pandemic national lockdown was implemented before we started to recruit to the planned 3-arm trial. Restrictions in CAMHS services due to lockdown meant that we could not offer face-to-face CT-PTSD. Therefore, after consultation with the funder and the TSC we changed the design to the current 2-arm trial and received HRA and REC approval to proceed. This change was made before recruitment started, and before registration on ISRCTN.

Further protocol amendments will require approval from the REC, and where relevant will be passed on to the trial register.

Consent and assent

For participants aged under 16, informed consent will be provided by carers and the young person will be asked for their assent. Participants aged 16 years or older can provide informed

For peer review only - http://bmjopen.bmj.com/site/about/guidelines.xhtml

consent without their parent or caregiver's involvement. Please see supplementary files for copies of consent and assent forms.

Confidentiality

Information with regards to participants will be kept confidential. The treating clinician and research team involved in day-to-day trial management will have access to personally identifiable data so that they can maintain contact with participants throughout the study. Participants will be assigned a study ID. All outcome data will be stored against this study ID so that data is anonymised.

Access to data

All investigators will have access to the final trial dataset. Our intentions are to maximise the availability and sharing of our data for the benefit of the wider research community, while providing for its long-term preservation and making due allowance for the potential commercial value of findings. The PMG will make the decision on whether to supply research data to a potential new researcher. Independent oversight of data access and sharing will be provided by the TSC. Data released to the wider community after publication will be fully anonymised.

Dissemination policy

There are no publication restrictions and findings will be disseminated broadly to participants, healthcare professionals, the public, and other relevant groups. The study findings will be published in peer-reviewed journals. The full trial protocol is available from PS.

For peer review only - http://bmjopen.bmj.com/site/about/guidelines.xhtml

DISCUSSION

PTSD in children and adolescents is a significant public health burden. Highly efficacious treatments exist but are not widely accessible. Remotely delivered iCT-PTSD has potential to facilitate a step change in improving accessibility of an evidence-based therapy for youth. The data gathered in the current trial will inform the design and size of a future scaled up trial to evaluate remotely delivered iCT-PTSD-YP.

For peer review only

For peer review only - http://bmjopen.bmj.com/site/about/guidelines.xhtml

Authors' contributions:

PS, DMC, TD, AE, KG, RMS, & WY designed the trial.

PS, DMC, TD, AE, HG, MG, DK, RMS, SM, DTP & WY contributed to App development and delivery

PS, DMC, EC, TD, AE, GF, KG, HG, DK, RMS, SM, & WY oversaw recruitment and data collection.

PS drafted the protocol.

All authors read and approved the final manuscript. All authors have agreed both to be personally accountable for their own contributions and to ensure that questions related to the accuracy or integrity of any part of the work, even ones in which the author was not personally involved, are appropriately investigated, resolved, and the resolution documented in the literature.

Declaration of interests

Some authors (DMC, TD, AE, RMS, DP, PS) provide training in the delivery of CT-PTSD, for which they may sometimes receive payment. PS, DMC, and WY are co-authors on a published treatment manual of CT-PTSD for children and young people [29] and receive royalties from sales.

Funding

OPTYC is funded by the UK Medical Research Council's Developmental Pathway Funding Scheme (Grant reference: MR/P017355/1). TD was funded by the UK Medical Research Council (Grant Reference: SUAG/043 G101400). KG receives funding from the NIHR Applied Research Collaboration South London (King's College Hospital NHS Foundation Trust).

For peer review only - http://bmjopen.bmj.com/site/about/guidelines.xhtml

REFERENCES

1. Copeland, W. E., Keeler, G., Angold, A., et al. (2007). Traumatic Events and Posttraumatic Stress in Childhood. *Arch Gen Psychiatry*, 64, 577-584.

2. Landolt, M.A., Schnyder, U., Maier, T., et al. (2013). Trauma exposure and posttraumatic stress disorder in adolescents: A national survey in Switzerland. *Journal of Traumatic Stress*. Vol.26(2), 209-216

3. McLaughlin, K. A., Koenen, K. C., Hill, E. D., et al. (2013). Trauma exposure and posttraumatic stress disorder in a national sample of adolescents. *J Am Acad Child Adolesc Psychiatry*, 52(8), 815-830

4. Morgan, L., Scourfield, J., Williams, D. et al (2003). The Aberfan disaster: 33 year follow up of survivors. *B J Psychiat*, 182, 6, 532-36

5. Yule, W., Bolton, D., Udwin, O., et al (2000). The long term psychological effects of a disaster experienced in adolescence I: incidence and course of PTSD. *J Child Psychiat & Psychol*. 41, 4, 503-11.

6. Morina, N., Koerssen, R., & Pollet, T. (2016). Interventions for children and adolescents with posttraumatic stress disorder: A meta-analysis of comparative outcome studies. *Clinical Psychology Review* 47 (2016) 41–54

For peer review only - http://bmjopen.bmj.com/site/about/guidelines.xhtml

7. Gutermann, J., Schreiber, F., Matulis, et al. (2016). Psychological Treatments for Symptoms of Posttraumatic Stress Disorder in Children, Adolescents, and Young Adults: A Meta-Analysis. *Clin Child Fam Psychol Rev*, 19(2), 77-93

8. Ehlers, A., & Clark, D. (2000). A cognitive model of posttraumatic stress disorder. *Behav Res Ther*, 38(4), 319–345.

9. Ehlers, A., Clark, D.M., Hackmann, A., et al. (2005). Cognitive therapy for PTSD: development and evaluation. *Behav Res Ther*, *43*, 413-431.

10. National Institute for Health and Care Excellence (2018). *Posttraumatic stress disorder. NICE guideline 116. https://www.nice.org.uk/guidance/ng116/resources*

11. Smith, P., Yule, W., Perrin, S., et al. (2007) Cognitive Behavior Therapy for PTSD in Children and Adolescents: A Randomized Controlled Trial. *J Am Acad Child and Adol Psychiat*, 46, 1051–1061.

12. Meiser-Stedman, R., Smith, P., McKinnon, A., et al. (2017). Cognitive therapy as an early treatment for post-traumatic stress disorder in children and adolescents: a randomized controlled trial addressing preliminary efficacy and mechanisms of action. *Journal of Child Psychology and Psychiatry*, 58(5), 623-633.

13. Mavranezouli, I., Megnin-Viggars, O., Daly, C., et al. (2019). Research Review: Psychological and psychosocial treatments for children and young people with post-traumatic stress disorder: a network meta-analysis. *Journal of Child Psychology and Psychiatry*, 61(1), 18-29.

For peer review only

For peer review only - http://bmjopen.bmj.com/site/about/guidelines.xhtml

14. Ford, T. Goodman, R., & Meltzer, H. (2003). Service use over 18 months among a nationally representative sample of British children with psychiatric disorder. *Clinical Child Psychology and Psychiatry*. 8, 1, 37-51.

15. Lewis, S., Arseneault L., Caspi, A., et al. (2019). The epidemiology of trauma and post-traumatic stress disorder in a representative cohort of young people in England and Wales. *Lancet Psychiatry*. Vol. 6 (3), 247-256

16. Care Quality Commission (2017). Review of children and young people's mental health services. Phase one report. CQC.

17. Hollis, C., Livingstone, S., & Sonuga-Barke, E. (2020). Editorial: The role of digital technology in children and young people's mental health – a triple-edged sword? *Journal of Child Psychology and Psychiatry*, 61(8), 837-41.

18. National Collaborating Centre for Mental Health (2014). E-therapies systematic review for children and young people with mental health problems. NCCMH, UK

19. Hollis, C., Falconer, C.J., Martin, J.L., et al. (2017). Annual Research Review: Digital health interventions for children and young people with mental health problems – a systematic and meta-review. *Journal of Child Psychology and Psychiatry*, 58(4), 474-503.

20. Christ, C., Schouten, M., Blankers, M., et al. (2020). Internet and computer-based cognitive behavioral therapy for anxiety and depression in adolescents and young adults: Systematic review and meta-analysis. *J Medical Internet Research*. 22, 9, e17831.

For peer review only

21. National Institute for Clinical Excellence (2019). Depression in children and young people: identification and management. London: NICE.

22. Bevan Jones, R., Stallard, P., Agha, S., et al. (2020). Practitioner review: Co-design of digital mental health technologies with children and young people. *Journal of Child Psychology and Psychiatry*, 61(8), 928-940.

23. Jaycox, L., Ayer, L., Vona, P., et al. (2019). Development and preliminary evaluation of a self-guided, internet-based tool for coping with stress and trauma: Life improvement for teens (LIFT). *Psychological Services*, 16, 1, 85-94

24. Kassam-Adams, N., Marsac, M., Kohser, K., et al. (2016). Pilot Randomized Controlled Trial of a Novel Web-Based Intervention to Prevent Posttraumatic Stress in Children Following Medical Events. *Journal of Pediatric Psychology*. 41(1):138-148.

25. Ruggiero, K., Price, M., Adams, Z., et al. (2015). Web Intervention for Adolescents Affected by Disaster: Population-Based Randomized Controlled Trial. *Journal of the American Academy of Child & Adolescent Psychiatry*. 54(9):709–717.

26. Sijbrandij M, Kunovski I, & Cuijpers P. (2016). Effectiveness of Internet-delivered cognitive behavioral therapy for posttraumatic stress disorder: a systematic review and meta-analysis. *Depression and Anxiety*, 33:783–91.

27. Smith, P., Perrin, S., Yule, W., et al. (2010). *Cognitive therapy for post- traumatic stress disorder*. Routledge Press, London

For peer review only - http://bmjopen.bmj.com/site/about/guidelines.xhtml

28. Ehlers, A., Wild, J., Warnock-Parkes, E., et al. (2020). Randomised controlled trial of therapist-assisted online psychological therapies for post-traumatic stress disorder (STOP-PTSD): trial protocol. *Trials*, 21:355. doi: 10.1186/s13063-020-4176-8

29. Wild, J., Warnock-Parkes, E., Grey, N., et al. (2016). Internet-delivered cognitive therapy for PTSD: a development pilot series, *European J Psychotrauma,* 7: 31019. doi:10.3402/ejpt.v7.310197

30. Hiller, R., Meiser-Stedman, R., Fearon, P., et al. (2016). Research Review: Changes in the prevalence and symptom severity of child post-traumatic stress disorder in the year following trauma – a meta-analytic study. *J Child Psychiat & Psychol*. 57, 8, 884-898.

31. Pynoos, R. S., Weathers, F. W., Steinberg, A. M., et al. (2015). *Clinician-Administered PTSD Scale for DSM-5 - Child/Adolescent Version*

32. Foa, E.B., Asnaani, A., Zang, Y., Capaldi, S., & Yen, R. (2018). Psychometrics of the child PTSD symptom scale (CPSS) for DSM-5 for trauma-exposed children and adolescents. Journal of Clinical Child and Adolescent Psychology, 47, 33-46

33. Dyregrov, A., & Yule, W. (1995). Screening measures: The development of the UNICEF screening battery. In annual meeting of the International Society for Traumatic Stress Studies, Boston, MA.

34. Perrin, S., Meiser-Stedman, R., & Smith, P. (2005). The Children's Revised Impact of Event Scale (CRIES): validity as a screening instrument for PTSD. *Behavioural and Cognitive Psychotherapy*, 33(4), 487-498.

For peer review only - http://bmjopen.bmj.com/site/about/guidelines.xhtml

35. Chorpita, B. F., Yim, L., Moffitt, C., Umemoto, L.A., & Francis, S.E. (2000). Assessment of symptoms of DSM-IV anxiety and depression in children: a revised child anxiety and depression scale. *Behaviour Research & Therapy,* 38, 835-855.

36. Goodman, R. (2001). Psychometric properties of the strengths and difficulties questionnaire. *Journal of the American Academy of Child and Adolescent Psychiatry*, 40 (11), 1337-1345.

37. McKinnon, A., Smith, P., Bryant, R., et al. (2016). An update on the clinical utility of the children's post-traumatic cognitions inventory. *Journal of Traumatic Stress*, 29(3), 253-258.

38. Meiser-Stedman, R., Smith, P., Yule, W. et al. (2007). The Trauma Memory Quality Questionnaire: Preliminary development and validation of a measure of trauma memory characteristics for children and adolescents. *Memory*, 15(3), 271-279.

39. Meiser-Stedman, R., Shepperd, A., Glucksman, E., et al. (2014). Thought control strategies and rumination in youth with acute stress disorder and posttraumatic stress disorder following single-event trauma. *Journal of Child and Adolescent Psychopharmacology*, 24(1), 47–51.

40. Stevens, K. (2012). Valuation of the child health utility 9D index. *Pharmacoeconomics*, 30(8), 729-747.

41. Shearer, J., Papanikolaou, N., Meiser-Stedman, R., et al. (2018). Cost-effectiveness of cognitive therapy as an early intervention for PTSD in children and adolescents: a trial-based evaluation and model. *Journal of Child Psychology and Psychiatry*, 59(7), 773-780.

For peer review only - http://bmjopen.bmj.com/site/about/guidelines.xhtml

42. Eldridge, S. M., Lancaster, G. A., Campbell, M. J., et al. (2016). Defining feasibility and pilot studies in preparation for randomised controlled trials: development of a conceptual framework. *PloS One*, 11(3), e0150205.

43. Thabane, L., Hopewell, S., Lancaster, G.A., et al. (2016). Methods and processes for development of a CONSORT extension for reporting pilot randomized controlled trials. *Pilot Feasibility Studies*, 20;2:25.

44. Vanderweele, T.J., & Vansteelandt, S. (2010). Odds Ratios for Mediation Analysis for a Dichotomous Outcome. *Am J Epidemiol*, 172(12):1339–48.

45. Valeri, L., & Vanderweele, T,J. (2013). Mediation analysis allowing for exposure-mediator interactions and causal interpretation: theoretical assumptions and implementation with SAS and SPSS macros. *Psychol Methods*, 18(2):137–50.

46. Fritz, M. S., Taylor, A. B., & MacKinnon, D. P. (2012). Explanation of two anomalous results in statistical mediation analysis. *Multivariate Behavioral Research*, 47(1), 61-87.

For peer review only - http://bmjopen.bmj.com/site/about/guidelines.xhtml

Acknowledgements

We are very grateful to the young people who helped to shape the project and made key contributions to the design of the intervention, and to the young people and carers who participate in the trial.

We are very grateful to the Trial Steering Committee (Cathy Creswell, Andrew Brand, Rachel Calam, & Paul Stallard) for their advice and support.

EC, GF and KG's contributions represent independent research part funded by the NIHR Biomedical Research Centre (South London and Maudsley NHS Foundation Trust and King's College London). KG receives funding from the NIHR Applied Research Collaboration South London (King's College Hospital NHS Foundation Trust). The views expressed are those of the authors and not necessarily those of the NHS, the NIHR, the Department of Health and Social Care.

Figure 1 Study flowchart

For peer review only - http://bmjopen.bmj.com/site/about/guidelines.xhtml

Figure 1 Study flowchart

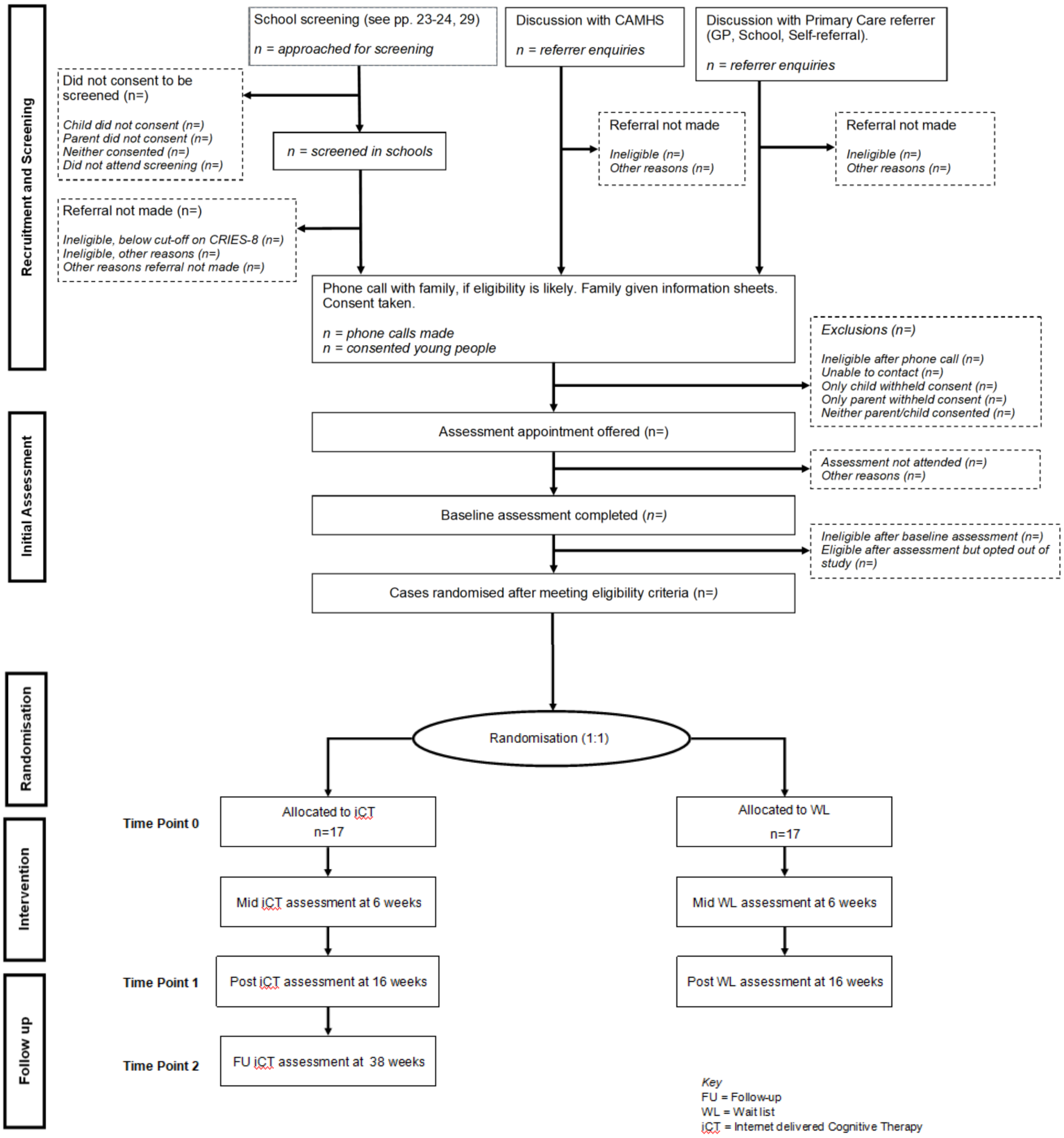

Checklist: World Health Organization Trial Registration Data Set

Data category	Information
Primary registry and trial identifying number	ISRCTN 16876240
Date of registration in primary registry	06/07/20
Secondary identifying numbers	N/A
Source(s) of monetary or material support	Medical Research Council UK
Primary sponsor	Joint Institute of Psychiatry, Psychology & Neuroscience and the South London and Maudsley NHS Foundation Trust.
Secondary sponsor(s)	N/A
Contact for public queries	Dr Patrick Smith, 020 7848 0506, patrick.smith@kcl.ac.uk
Contact for scientific queries	Dr Patrick Smith, as above
Public title	Online post-traumatic stress disorder treatment for young people and their carers
Scientific title	As above
Countries of recruitment	UK
Health condition(s) or problem(s) studied	Post-Traumatic Stress Disorder (PTSD)
Intervention(s)	Internet delivered Cognitive Therapy for PTSD in Young People (iCT-PTSD-YP)
Key inclusion and exclusion criteria	Young people:<br>1. Aged 12-17 years old<br>2. Main presenting problem is PTSD (diagnosed using CAPS-5-CA) and there is a not a co-morbid problem that would preclude treatment of PTSD<br>3. PTSD symptoms relate to a single trauma<br>4. Participant has access to compatible smartphone or larger computing device (e.g. laptop, desktop computer, iPad) with internet access and to a safe and confidential space in which to engage in iCT<br>5. Participant speaks English to a level that allows therapy without the need for an interpreter, and reads English to a level that allows independent use of iCT<br><br>Parents or carers:<br>1. Parent or carer of a young person who meets all of the inclusion criteria above and none of the exclusion criteria below<br>2. Parent or carer speaks English to a level that allows participation in therapy without the need for an interpreter, and reads English to a level that allows independent use of iCT<br>3. Parent or carer has access to compatible smartphone or larger computing device (e.g. laptop, desktop computer, iPad) with internet access

For peer review only

Study type	Two-arm parallel-group single-blind (outcome assessor) early-stage randomized controlled trial
Date of first enrolment	25/08/20
Target sample size	34
Recruitment status	recruiting
Primary outcome(s)	Feasibility<br>As this is an early-stage trial, the primary outcomes are feasibility outcomes and adherence metrics. Feasibility data on acceptability, compliance, retention, and delivery will be collected.<br><br>Clinical<br>The primary clinical outcome is presence or absence of PTSD 16 weeks after randomisation, determined by administration of a gold standard semi-structured interview by a trained reliable assessor who is blind to treatment allocation.
Key secondary outcomes	Secondary clinical outcomes are continuous scores on a battery of reliable and valid questionnaires measuring severity of PTSD, anxiety, and depression, completed by young people and carers.

For peer review only

Online PTSD Treatment for Young People & Carers

PARTICIPANT CONSENT FORM

This consent form is for young people aged 16+

Online PTSD treatment for Young People and their Carers (OPTYC): RCT
Dr Patrick Smith

1. I confirm that I have read the information sheet dated 06/05/2020 (version 1.2) for the above study. I have had the opportunity to consider the information, ask questions and have had these answered satisfactorily. ☐
2. I understand that my participation is voluntary and that I am free to withdraw at any time without giving any reason, without my medical care or legal rights being affected. ☐
3. I understand that relevant data collected during the study may be looked at by individuals from King's College London, from regulatory authorities or from the NHS Trust, where it is relevant to my taking part in this research. I give permission for these individuals to have access to my records. ☐
4. I understand that the information collected about me will be used to support other research in the future, and may be shared anonymously with other researchers. ☐
5. I understand that my relevant confidential information will be disclosed to appropriate professionals, including my GP, if a clinical or research worker on the study becomes concerned about my own, or someone else's safety. ☐
6. I agree to my General Practitioner being informed of my participation in the study and being involved in the study, including any necessary exchange of information about me between my GP and the research team. ☐
7. I understand that the information held and maintained by South London and Maudsley NHS Foundation Trust [to be localised] may be used to help contact me or provide information about my health status. ☐
8. I consent to the recording of an interview with me being made and kept on videotape/audiotape. I understand that this recording may be used for purposes of this research project.
9. I agree to take part in the above study. ☐

____________________	____________	________________
Name of Participant	Date	Signature
____________________	____________	________________
Name of Person taking consent	Date	Signature

Online PTSD Treatment for Young People & Carers

PARTICIPANT ASSENT FORM

This assent form is for young people aged 12-15

Please complete this form after you have read the Information Sheet or listened to an explanation about the research.

Online PTSD treatment for Young People and their Carers (OPTYC): RCT
Dr Patrick Smith

1. I confirm that I have read the information sheet dated 06/05/2020 (version 1.2) for the above study. I have had the opportunity to consider the information, ask questions and have had these answered satisfactorily. ☐

2. I understand that my participation is voluntary. If I decide at any time during the research that I no longer wish to take part, I can tell the researchers and pull out and I don't have to give a reason. If I pull out it will not affect my medical care or legal rights. ☐

3. I understand that relevant data collected during the study may be looked at by individuals from King's College London, from regulatory authorities or from the NHS Trust, where it is relevant to my taking part in this research. I give permission for these individuals to have access to my records. ☐

4. I understand that the information collected about me will be used to support other research in the future, and may be shared anonymously with other researchers. ☐

5. I understand that my relevant confidential information will be disclosed to appropriate professionals, including my GP, if a clinical or research worker on the study becomes concerned about my own, or someone else's safety. ☐

6. I agree to my General Practitioner being informed of my participation in the study and being involved in the study, including any necessary exchange of information about me between my GP and the research team. ☐

7. I understand that the information held and maintained by South London and Maudsley NHS Foundation Trust [to be localised] may be used to help contact me or provide information about my health status. ☐

8. I agree that an interview with me can be recorded and kept on videotape/audiotape. I understand that this recording may be used for purposes of this research project. ☐

9. I understand that because I am under 16 years old, I can provide my informed assent to take part in this study, but my parent/carer will also need to provide formal consent for me to take part. I agree to take part in the above study. ☐

______________________________	_______________	____________________
Name of Participant	Date	Signature
______________________________	_______________	____________________
Name of Person taking consent	Date	Signature

Online PTSD Treatment for Young People & Carers

PARENTAL CONSENT FORM

This consent form is for parents/carers of participants aged 12-15

Online PTSD treatment for Young People and their Carers (OPTYC): RCT
Dr Patrick Smith

1. I confirm that I have read the information sheet v1.2 dated 06.05.2020 for the above study. I have been consulted about my child's participation in this research project. I have had the opportunity to ask questions about the study and understand what is involved. I agree to their taking part in this research. ☐

2. I understand that my child's participation is voluntary and that I can request that they are withdrawn from the study at any time without giving any reason, and without their medical care or legal rights being affected. ☐

3. I understand that relevant data collected during the study about my child, may be looked at by individuals from King's College London, from regulatory authorities or from the NHS Trust, where it is relevant to their taking part in this research. I give permission for these individuals to have access to my child's records. ☐

4. I understand that the information collected about my child will be used to support other research in the future, and may be shared anonymously with other researchers. ☐

5. I understand that my child's relevant confidential information will be disclosed to appropriate professionals, including their GP, if a clinical or research worker on the study becomes concerned about my child's, or someone else's safety. ☐

6. I agree to my child's General Practitioner being informed of their participation in the study and being involved in the study, including any necessary exchange of information about them between their GP and the research team. ☐

7. I understand that the information held and maintained by South London and Maudsley NHS Foundation Trust [to be localised] may be used to help contact my child or provide information about their health status. ☐

8. I agree for my child's assessment, and if relevant their treatment sessions, to be audio/video recorded. I understand that this recording may be used for the purposes of this research project. ☐

9. I agree for my child to take part in the above study. ☐

______________________	______________________	______________	__________________
Your Name	Relationship to child	Date	Signature

Online PTSD Treatment for Young People & Carers

Name of Person taking consent	Date	Signature

Online PTSD Treatment for Young People & Carers

PARTICIPANT CONSENT FORM FOR PARENTS/CARERS

This consent form is for <u>parents/carers who wish to take part in the study</u>

Online PTSD treatment for Young People and their Carers (OPTYC): RCT
Dr Patrick Smith

1. I confirm that I have read the information sheet dated 06/05/2020 (version 1.2) for the above study. I have had the opportunity to consider the information, ask questions and have had these answered satisfactorily. ☐
2. I understand that my participation is voluntary and that I am free to withdraw at any time without giving any reason, without my medical care or legal rights being affected. ☐
3. I understand that relevant data collected during the study may be looked at by individuals from King's College London, from regulatory authorities or from the NHS Trust, where it is relevant to my taking part in this research. I give permission for these individuals to have access to my records. ☐
4. I understand that the information collected about me will be used to support other research in the future, and may be shared anonymously with other researchers. ☐
5. I understand that the information held and maintained by South London and Maudsley NHS Foundation Trust [to be localised] may be used to help contact me or provide information about my health status. ☐
6. I understand that my relevant confidential information will be disclosed to appropriate professionals, including my GP, if a clinical or research worker on the study becomes concerned about my own, or someone else's safety. ☐
7. I consent to the recording of an interview with me being made and kept on videotape/audiotape. I understand that this recording may be used for purposes of this research project. ☐
8. I agree to take part in the above study. ☐

____________________	____________________	____________________
Name of Participant	Date	Signature

____________________	____________________	____________________
Name of Person taking consent	Date	Signature

PARTICIPANT CONSENT FORM

This consent form is for young people aged 16+

Online PTSD treatment for Young People and their Carers (OPTYC): School Screening

Dr Patrick Smith

Please initial box

1. I confirm that I have read the information sheet dated 06.05.2020 (version 1.2) for the school screening for the OPTYC study. I have had the opportunity to consider the information, ask questions and have had these answered satisfactorily. ☐

2. I understand that my participation is voluntary and that I am free to withdraw at any time without giving any reason, without my medical care or legal rights being affected. ☐

3. I understand that relevant data collected during the study may be looked at by individuals from King's College London, from regulatory authorities or from the NHS Trust, where it is relevant to my taking part in this research. I give permission for these individuals to have access to my records. ☐

4. I understand that the information collected about me will be used to support other research in the future and may be shared **anonymously** with other researchers. ☐

5. I understand that my relevant confidential information will be disclosed to my parent/carer and appropriate professionals, including my General Practitioner (GP), if a clinical or research worker on the study becomes concerned about my own, or someone else's safety. ☐

6. I agree to my GP being informed of my participation in the study, including any necessary exchange of information about me between my GP and the research team. ☐

7. I agree to take part in the school screening part of this study. ☐

8. I consent to you contacting me via the details provided below. ☐

PLEASE TURN OVER

Online PTSD Treatment for Young People & Carers

Your Name ____

Your School and Form ____

Your Contact Telephone Number ___________________________________

Your Home Address ____

Your Email Address ____

___________________________ ________________ ______________________

Name of Participant Date Signature

PLEASE RETURN THIS FORM TO YOUR FORM TUTOR / SCHOOL RECEPTION STAFF / VIA THE PROVIDED FREEPOST ENVELOPE

To be signed by member of OPTYC Team:

______________________________ __________________ ______________________

Name of Person taking consent Date Signature

Online PTSD Treatment for Young People & Carers

don’t have to give a reason. I understand that if I pull out it will not affect my medical

individuals from King’s College London, from regulatory authorities or from the NHS

someone else’s safety.

OPTYC
Online PTSD Treatment for Young People & Carers

King's College London

MRC Medical Research Council

NHS South London and Maudsley NHS Foundation Trust

Online PTSD Treatment for Young People & Carers

PARENTAL CONSENT FORM

This consent form is for parents/carers of participants aged 12-15

Online PTSD treatment for Young People and their Carers (OPTYC): School Screening

Dr Patrick Smith

1. I confirm that I have been consulted about my child's participation in the '**school screening**' part of this research project. I have read the information sheet dated 06.05.2020 (version 1.2) for the school screening and have had the opportunity to ask questions about the study and understand what is involved. ☐

2. I understand that my child's participation is voluntary and that I can request that they are withdrawn from the study at any time without giving any reason, and without their medical care or legal rights being affected. ☐

3. I understand that relevant data collected during the study about my child, may be looked at by individuals from King's College London, from regulatory authorities or from the NHS Trust, where it is relevant to their taking part in this research. I give permission for these individuals to have access to my child's records. ☐

4. I understand that the information collected about my child will be used to support other research in the future and may be shared **anonymously** with other researchers. ☐

5. I understand that my child's relevant confidential information will be disclosed to appropriate professionals, including their General Practitioner (GP), if a clinical or research worker on the study becomes concerned about my child's, or someone else's safety. ☐

6. I agree to my child's GP being informed of their participation in the study, including any necessary exchange of information about them between their GP and the research team. ☐

7. I agree for my child to take part in the school screening part of this study. ☐

8. I consent to you contacting my child and me via the details provided below. ☐

PLEASE TURN OVER

Online PTSD Treatment for Young People & Carers

Your Child's Name ____________________

Your Child's School and Form ____________________

Your Child's Contact Telephone Number ____________________

Your Child's Email Address ____________________

Your Contact Telephone Number ____________________

Your Home Address ____________________

Your Email Address ____________________

Your Name	Relationship to child	Date	Signature

PLEASE RETURN THIS FORM TO YOUR CHILD'S FORM TUTOR / SCHOOL RECEPTION STAFF/ VIA THE PROVIDED PRE-PAID ENVELOPE

To be signed by member of OPTYC Team:

Name of Person taking consent	Date	Signature

Reporting checklist for protocol of a clinical trial.

Based on the SPIRIT guidelines.

Instructions to authors

Complete this checklist by entering the page numbers from your manuscript where readers will find each of the items listed below.

Your article may not currently address all the items on the checklist. Please modify your text to include the missing information. If you are certain that an item does not apply, please write "n/a" and provide a short explanation.

Upload your completed checklist as an extra file when you submit to a journal.

In your methods section, say that you used the SPIRITreporting guidelines, and cite them as:

Chan A-W, Tetzlaff JM, Gøtzsche PC, Altman DG, Mann H, Berlin J, Dickersin K, Hróbjartsson A, Schulz KF, Parulekar WR, Krleža-Jerić K, Laupacis A, Moher D. SPIRIT 2013 Explanation and Elaboration: Guidance for protocols of clinical trials. BMJ. 2013;346:e7586

		Reporting Item	Page Number
**Administrative information**			
Title	#1	Descriptive title identifying the study design, population, interventions, and, if applicable, trial acronym	1
Trial registration	#2a	Trial identifier and registry name. If not yet registered, name of intended registry	3
Trial registration: data set	#2b	All items from the World Health Organization Trial Registration Data Set	3, Appendix 1
Protocol version	#3	Date and version identifier	4
Funding	#4	Sources and types of financial, material, and other support	26
Roles and responsibilities: contributorship	#5a	Names, affiliations, and roles of protocol contributors	1, 26

For peer review only

Roles and responsibilities: sponsor contact information	#5b	Name and contact information for the trial sponsor	5
Roles and responsibilities: sponsor and funder	#5c	Role of study sponsor and funders, if any, in study design; collection, management, analysis, and interpretation of data; writing of the report; and the decision to submit the report for publication, including whether they will have ultimate authority over any of these activities	5
Roles and responsibilities: committees	#5d	Composition, roles, and responsibilities of the coordinating centre, steering committee, endpoint adjudication committee, data management team, and other individuals or groups overseeing the trial, if applicable (see Item 21a for data monitoring committee)	22
**Introduction**			
Background and rationale	#6a	Description of research question and justification for undertaking the trial, including summary of relevant studies (published and unpublished) examining benefits and harms for each intervention	6
Background and rationale: choice of comparators	#6b	Explanation for choice of comparators	11-12
Objectives	#7	Specific objectives or hypotheses	8
Trial design	#8	Description of trial design including type of trial (eg, parallel group, crossover, factorial, single group), allocation ratio, and framework (eg, superiority, equivalence, non-inferiority, exploratory)	8
**Methods: Participants, interventions, and outcomes**			

For peer review only

Study setting	#9	Description of study settings (eg, community clinic, academic hospital) and list of countries where data will be collected. Reference to where list of study sites can be obtained	9
Eligibility criteria	#10	Inclusion and exclusion criteria for participants. If applicable, eligibility criteria for study centres and individuals who will perform the interventions (eg, surgeons, psychotherapists)	9, 10
Interventions: description	#11a	Interventions for each group with sufficient detail to allow replication, including how and when they will be administered	10-11
Interventions: modifications	#11b	Criteria for discontinuing or modifying allocated interventions for a given trial participant (eg, drug dose change in response to harms, participant request, or improving / worsening disease)	12
Interventions: adherence	#11c	Strategies to improve adherence to intervention protocols, and any procedures for monitoring adherence (eg, drug tablet return; laboratory tests)	13
Interventions: concomitant care	#11d	Relevant concomitant care and interventions that are permitted or prohibited during the trial	9
Outcomes	#12	Primary, secondary, and other outcomes, including the specific measurement variable (eg, systolic blood pressure), analysis metric (eg, change from baseline, final value, time to event), method of aggregation (eg, median, proportion), and time point for each outcome. Explanation of the clinical relevance of chosen efficacy and harm outcomes is strongly recommended	12-17<br>table 1
Participant timeline	#13	Time schedule of enrolment, interventions (including any run-ins and washouts), assessments, and visits for participants. A schematic diagram is highly recommended (see Figure)	17

For peer review only

Sample size	#14	Estimated number of participants needed to achieve study objectives and how it was determined, including clinical and statistical assumptions supporting any sample size calculations	17
Recruitment	#15	Strategies for achieving adequate participant enrolment to reach target sample size	18
**Methods: Assignment of interventions (for controlled trials)**			
Allocation: sequence generation	#16a	Method of generating the allocation sequence (eg, computer-generated random numbers), and list of any factors for stratification. To reduce predictability of a random sequence, details of any planned restriction (eg, blocking) should be provided in a separate document that is unavailable to those who enrol participants or assign interventions	18
Allocation concealment mechanism	#16b	Mechanism of implementing the allocation sequence (eg, central telephone; sequentially numbered, opaque, sealed envelopes), describing any steps to conceal the sequence until interventions are assigned	18
Allocation: implementation	#16c	Who will generate the allocation sequence, who will enrol participants, and who will assign participants to interventions	18
Blinding (masking)	#17a	Who will be blinded after assignment to interventions (eg, trial participants, care providers, outcome assessors, data analysts), and how	18
Blinding (masking): emergency unblinding	#17b	If blinded, circumstances under which unblinding is permissible, and procedure for revealing a participant's allocated intervention during the trial	n/a

For peer review only

**Methods: Data collection, management, and analysis**			
Data collection plan	#18a	Plans for assessment and collection of outcome, baseline, and other trial data, including any related processes to promote data quality (eg, duplicate measurements, training of assessors) and a description of study instruments (eg, questionnaires, laboratory tests) along with their reliability and validity, if known. Reference to where data collection forms can be found, if not in the protocol	19
Data collection plan: retention	#18b	Plans to promote participant retention and complete follow-up, including list of any outcome data to be collected for participants who discontinue or deviate from intervention protocols	12, 19
Data management	#19	Plans for data entry, coding, security, and storage, including any related processes to promote data quality (eg, double data entry; range checks for data values). Reference to where details of data management procedures can be found, if not in the protocol	19
Statistics: outcomes	#20a	Statistical methods for analysing primary and secondary outcomes. Reference to where other details of the statistical analysis plan can be found, if not in the protocol	19-20
Statistics: additional analyses	#20b	Methods for any additional analyses (eg, subgroup and adjusted analyses)	19 - 22
Statistics: analysis population and missing data	#20c	Definition of analysis population relating to protocol non-adherence (eg, as randomised analysis), and any statistical methods to handle missing data (eg, multiple imputation)	20, 21
**Methods: Monitoring**			

For peer review only

Data monitoring: formal committee	#21a	Composition of data monitoring committee (DMC); summary of its role and reporting structure; statement of whether it is independent from the sponsor and competing interests; and reference to where further details about its charter can be found, if not in the protocol. Alternatively, an explanation of why a DMC is not needed	22
Data monitoring: interim analysis	#21b	Description of any interim analyses and stopping guidelines, including who will have access to these interim results and make the final decision to terminate the trial	20
Harms	#22	Plans for collecting, assessing, reporting, and managing solicited and spontaneously reported adverse events and other unintended effects of trial interventions or trial conduct	23
Auditing	#23	Frequency and procedures for auditing trial conduct, if any, and whether the process will be independent from investigators and the sponsor	n/a
**Ethics and dissemination**			
Research ethics approval	#24	Plans for seeking research ethics committee / institutional review board (REC / IRB) approval	23
Protocol amendments	#25	Plans for communicating important protocol modifications (eg, changes to eligibility criteria, outcomes, analyses) to relevant parties (eg, investigators, REC / IRBs, trial participants, trial registries, journals, regulators)	24
Consent or assent	#26a	Who will obtain informed consent or assent from potential trial participants or authorised surrogates, and how (see Item 32)	24
Consent or assent: ancillary studies	#26b	Additional consent provisions for collection and use of participant data and biological specimens in ancillary studies, if applicable	n/a

For peer review only

Confidentiality	#27	How personal information about potential and enrolled participants will be collected, shared, and maintained in order to protect confidentiality before, during, and after the trial	24
Declaration of interests	#28	Financial and other competing interests for principal investigators for the overall trial and each study site	26
Data access	#29	Statement of who will have access to the final trial dataset, and disclosure of contractual agreements that limit such access for investigators	25
Ancillary and post trial care	#30	Provisions, if any, for ancillary and post-trial care, and for compensation to those who suffer harm from trial participation	n/a
Dissemination policy: trial results	#31a	Plans for investigators and sponsor to communicate trial results to participants, healthcare professionals, the public, and other relevant groups (eg, via publication, reporting in results databases, or other data sharing arrangements), including any publication restrictions	25
Dissemination policy: authorship	#31b	Authorship eligibility guidelines and any intended use of professional writers	n/a
Dissemination policy: reproducible research	#31c	Plans, if any, for granting public access to the full protocol, participant-level dataset, and statistical code	25
**Appendices**			
Informed consent materials	#32	Model consent form and other related documentation given to participants and authorised surrogates	Supplementary file uploaded to BMJ site
Biological specimens	#33	Plans for collection, laboratory evaluation, and storage of biological specimens for genetic or molecular analysis in the current trial and for future use in ancillary studies, if applicable	n/a

For peer review only

None The SPIRIT Explanation and Elaboration paper is distributed under the terms of the Creative Commons Attribution License CC-BY-NC. This checklist can be completed online using https://www.goodreports.org/, a tool made by the EQUATOR Network in collaboration with Penelope.ai

For peer review only
